# Improved pea reference genome and pan-genome highlight genomic features and evolutionary characteristics

**DOI:** 10.1038/s41588-022-01172-2

**Published:** 2022-09-22

**Authors:** Tao Yang, Rong Liu, Yingfeng Luo, Songnian Hu, Dong Wang, Chenyu Wang, Manish K. Pandey, Song Ge, Quanle Xu, Nana Li, Guan Li, Yuning Huang, Rachit K. Saxena, Yishan Ji, Mengwei Li, Xin Yan, Yuhua He, Yujiao Liu, Xuejun Wang, Chao Xiang, Rajeev K. Varshney, Hanfeng Ding, Shenghan Gao, Xuxiao Zong

**Affiliations:** 1 National Key Facility for Crop Gene Resources and Genetic Improvement / Institute of Crop Sciences, Chinese Academy of Agricultural Sciences, Beijing, China; 2 State Key Laboratory of Microbial Resources, Institute of Microbiology, Chinese Academy of Sciences, Beijing, China; 3 Institute of Crop Germplasm Resources, Shandong Academy of Agricultural Sciences / Shandong Provincial Key Laboratory of Crop Genetic Improvement, Ecology and Physiology, Jinan, China; 4 Center of Excellence in Genomics & Systems Biology, International Crops Research Institute for the Semi-Arid Tropics (ICRISAT), Hyderabad, India; 5 State Key Laboratory of Systematic and Evolutionary Botany, Institute of Botany, Chinese Academy of Sciences, Beijing, China; 6 College of Life Sciences, Northwest A&F University, Yangling, China; 7 Institute of Grain Crops, Yunnan Academy of Agricultural Sciences, Kunming, China; 8 State Key Laboratory of Plateau Ecology and Agriculture, Qinghai University, Xining, China; 9 Qinghai Academy of Agricultural and Forestry Sciences, Xining, China; 10 Jiangsu Yanjiang Institute of Agricultural Sciences, Nantong, China; 11 Crop Research Institute, Sichuan Academy of Agricultural Sciences, Chengdu, China; 12 Murdoch’s Centre for Crop and Food Innovation, WA State Agricultural Biotechnology Centre, Food Futures Institute, Murdoch University, Murdoch, Western Australia Australia; 13 College of Life Science, Shandong Normal University, Jinan, China; 14 University of Chinese Academy of Sciences, Beijing, China

**Keywords:** Genomics, Plant breeding

## Abstract

Complete and accurate reference genomes and annotations provide fundamental resources for functional genomics and crop breeding. Here we report a de novo assembly and annotation of a pea cultivar ZW6 with contig N50 of 8.98 Mb, which features a 243-fold increase in contig length and evident improvements in the continuity and quality of sequence in complex repeat regions compared with the existing one. Genome diversity of 118 cultivated and wild pea demonstrated that *Pisum abyssinicum* is a separate species different from *P. fulvum* and *P. sativum* within *Pisum*. Quantitative trait locus analyses uncovered two known Mendel’s genes related to stem length (*Le/le*) and seed shape (*R/r*) as well as some candidate genes for pod form studied by Mendel. A pan-genome of 116 pea accessions was constructed, and pan-genes preferred in *P. abyssinicum* and *P. fulvum* showed distinct functional enrichment, indicating the potential value of them as pea breeding resources in the future.

## Abstract (web summary)

A high-quality genome assembly of pea cultivar ZW6 and pan-genome analyses provide insights into pea genome evolution and domestication as well as genomic resources for pea improvement.

## Main

Identifying and understanding the genetic basis of phenotypic variation during domestication is one of the major focus in modern genetics and evolutionary biology^1,2^. In the past decades, next-generation sequencing (NGS) technology has greatly facilitated crop genomics studies leading to a better understanding of genome architecture and complexity^3,4^. A high-quality reference genome and complete annotation provide important tools for population genomics and molecular genetics research to understand crop domestication and accelerate genetic improvement^5,6^. Numerous studies on crop population genomics and genome-wide association analyses based on single-nucleotide polymorphisms (SNPs) and small insertion/deletion (indel) polymorphisms have laid an important foundation for understanding crop domestication and gene mining of important traits^7–11^. Many studies have identified structural variations (SVs) involved in defining genome structure, gene function and expression levels and characterized their crucial roles in plant evolution, phenotypic diversity and crop improvement^12–16^. However, SV lengths, types, distribution and population frequency and their contribution to phenotypes have not been fully described^15,17,18^.

An increasing number of studies have proven that a single reference genome is insufficient to represent a species, particularly due to the diversification and alterations of genetic structure associated with the long-term domestication of crops, and pan-genomes constructed from diverse individuals are gaining popularity as a tool to capture the diversity within a species^9,14,16,19–22^. Recent studies on plant pan-genome have successfully uncovered the abundant presence/absence variations (PAVs) in functionally important genes, with the proportions of core genes/orthologous gene clusters ranging from 33% to 92%^21^. The discovery of large-scale SVs and their association with genome evolution, gene expression and agronomic traits have also been reported. Such studies have contributed to understanding crop domestication, exploring gene function and using breeding resources^18,21,22^.

Pea (*Pisum sativum* L., 2n = 2x = 14), an annual cool-season legume, belongs to Leguminosae, Papilionoideae and *Pisum* with a genome size of approximately 4.45 Gb^23,24^. Pea is a multifunctional crop in the food and feed industry as a fresh vegetable and dry grain^24,25^. The harvested area of peas are ranked fourth among legumes, after soybeans, common beans and chickpeas (http://www.fao.org/faostat/). As a source of protein, starch, fiber and minerals^26,27^ endowed with a notable ecological sustainability advantage due to its biological nitrogen fixation capacity^28^, pea has continued to draw attention, especially since Mendel uncovered the laws of inheritance through breeding experiments with peas^29,30^. Pea was inferred to have been domesticated by Neolithic farmers in the Near East and the Middle East approximately 10,000 years ago and is considered one of the earliest domesticated legume crops^31–33^. However, despite its critical role in advancing plant genetics, its domestication process remains a mystery, and the genetic diversity of cultivated and wild peas within *Pisum* has yet to be fully uncovered.

The recent availability of a reference genome for pea constructed based on NGS technology provided insights into legume genome evolution^34^. However, an improved genome assembly and genome annotation are required for a better understanding of the phenotypic variation and genome evolution of the pea^6,35,36^. This Article presents a de novo genome assembly of a pea cultivar, ZW6, that was constructed based on full PacBio single-molecule real-time (SMRT) sequencing in combination of 10x Genomics sequencing, Bionano optical mapping and chromosome conformation capture (Hi-C) sequencing, as well as Illumina NGS technologies. This assembly provides a evidently improved reference genome and annotation of pea. We further identified genome-wide variations (SNPs, indels and SVs) and present the population genetic structure of 118 cultivated and wild pea genotypes based on whole genome resequencing data. Through genome selection and quantitative trait locus (QTL) analyses, a batch of candidate genes related to domestication and breeding improvement traits, including several candidates for Mendel’s genes were discovered. We also report a pea pan-genome based on these 118 accessions that provide a large number of additional genes and sequences not present in the reference genome. The high-quality reference genome and pan-genome offer insights into pea genome evolution and domestication as well as valuable genomic resources for research in pea genetics and breeding^22,37^.

## Results

### Construction and evaluation of genome assembly PeaZW6

**Fig. 1:**
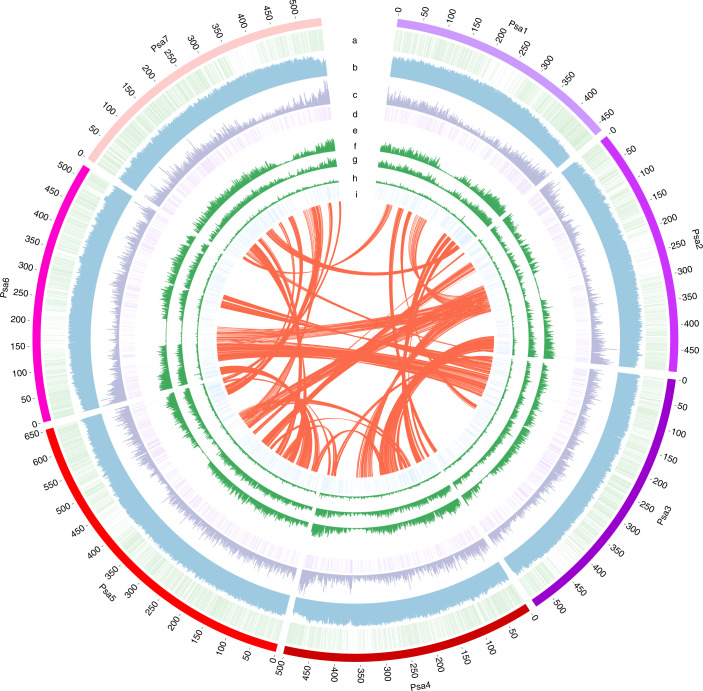
Overview of the pea genome assembly. The outer layer of colored blocks is a circular representation of seven chromosomes. a = the genetic markers, b = repeat density, c = gene density calculated in 1,000-kb windows sliding in 500-kb steps, d = tandem duplicated genes, e = Mendel’s genes (red lines); f, g and h = the nucleotide diversity (π) of the three species within *Pisum* (*P. sativum* (64), *P. fulvum* (22) and *P. abyssinicum* (15)) based on population genetic structure analyses, and i = transcription factors. The innermost layer shows interchromosomal synteny.

ZW6 is a widely grown Chinese pea cultivar (Supplementary Fig. 1). The estimated genome size of ZW6 was 4.28 Gb using flow cytometry (Supplementary Fig. 2 and Supplementary Table 1) and 4.26 Gb using K-mer analysis (Supplementary Fig. 3). These estimates are smaller than the previously reported genome size (4.45 Gb)^23,24^. K-mer analysis also showed a very low heterozygosity ratio (0.08%) and a high proportion of repeat sequences (83%) in ZW6 (Supplementary Fig. 3). Using a combination of PacBio SMRT sequencing, 10x Genomics scaffolding, Bionano optical mapping, Hi-C scaffolding and Illumina NGS technologies (Supplementary Fig. 4 and Supplementary Table 2), a high-quality, high-continuity chromosome-level reference assembly of ZW6 (PeaZW6) was constructed (Fig. 1 and Table 1). The initial assembly based on 379.34 Gb of PacBio reads (~85.2× genomic coverage) had a total size of 3,796.7 Mb and a contig N50 size of 8.98 Mb. After polishing, iterative scaffolding and manual curation (Supplementary Fig. 5), the final assembly was anchored into seven chromosome-level pseudomolecules, with two organelle genomes and 1,572 unplaced contigs (Fig. 1 and Table 1). The total size of anchored contigs was 3,719.6 Mb, constituting 97.96% of PeaZW6, whereas anchored contigs constituted only 82.51% of the previous NGS-based assembly of Caméor (PeaCaméor)^34^. The cumulative length of unknown sequences was 10.3 Mb, which was much smaller than 760.8 Mb in PeaCaméor^34^. After mapping the corrected reads to PeaZW6, it was found that 99.41% and 99.16% of the assembly was covered by at least 20 PacBio reads and 20 NGS reads, respectively, which confirmed the high quality of PeaZW6 (Supplementary Table 3, Supplementary Fig. 6 and Supplementary Notes).

**Table 1:** Summary of pea genome assembly

Genome feature	PeaZW6		PeaCaméor	
	Number	Size (Mb)	Number	Size(Mb)
Assembled scaffolds	1,579	3,796.7	24,623	3,920.1
Superscaffolds	7	3,719.6	7	3,234.4
Remaining scaffolds	1,572	77.1	14,266	685.4
N50 remaining scaffolds	289	0.074	1,411	0.13
Contigs	2,402	3,786.4	218,010	3,159.3
N50 contigs	118	8.98	32,663	0.037
Remaining contigs	1,586	76.6	69,733	572.1
N50 remaining contigs	298	0.073	6,466	0.023
Protein-coding gene models	47,526	121.84	44,756	124.6
Genes in pseudomolecules	46,607	119.68	38,312	102.3

The improved PeaZW6 also showed higher BUSCO completeness (99.38%, genome mode) than PeaCaméor (96.78%, genome mode) (Supplementary Table 4). The mapping rate of qualified RNA sequencing (RNA-seq) reads from most tissues was greater than 99% (Supplementary Table 5). In addition, Merqury analysis showed a nearly doubled consensus quality value (QV) of PeaZW6 (44.5) compared to PeaCaméor (24.3) (Supplementary Table 6), confirming the higher quality and greater accuracy of PeaZW6. Specifically, PeaZW6 harbored 98.5% uniquely mapped genetic markers, indicating a high level of collinearity between the chromosome-level assembly and the previous reported genetic map^38^ (Supplementary Fig. 7). Syntenic regions between genomes of pea and Medicago (*Medicago truncatula*) were detected and showed that the number of homologous genes within the syntenic regions of PeaZW6/Medicago was evidently and consistently greater than that in PeaCaméor/Medicago with different parameters (Supplementary Table 7 and Supplementary Notes), validating the long-range continuousness of PeaZW6.

### Genome annotation for PeaZW6

**Fig. 2:**
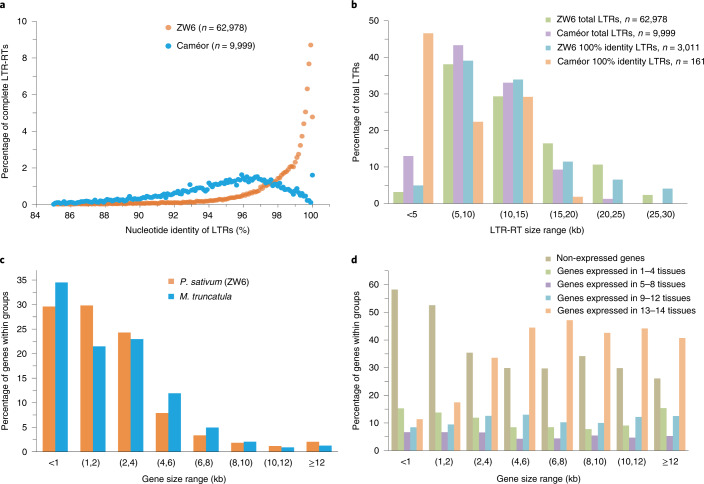
The comparative and functional characterization of repeats. **a**, Nucleotide identity distribution of long terminal region of the complete LTR retrotransposons. **b**, Length distribution of the complete LTR-RTs in pea genome. **c**, Comparison of gene length between *P. sativum* (ZW6) and *M. truncatula*. **d**, Expression characterization of pea genes with different length.

The total length of repetitive elements in PeaZW6 was 3,249.5 Mb, larger than that in PeaCaméor (2,662.5 Mb). Gypsy was the dominant type of transposable element, accounting for 54.34% of PeaZW6 (Supplementary Data 1). Long-terminal repeat (LTR) assembly index (LAI) analysis indicated a substantial improvement in LTR-retrotransposon (LTR-RT) completeness for PeaZW6 (LAI = 13.31) compared to PeaCaméor (LAI = 2.09) (Supplementary Table 8). PeaZW6 had many more full-length LTR-RTs than PeaCaméor and a higher percentage of active and longer LTRs (Fig. 2a,b). These results may explain the reasons for the obvious differences in gap size between the PacBio-based PeaZW6 (10.3 Mb) and the NGS-based PeaCaméor (760.8 Mb). The improved LTR-RT completeness indicated that the assembly of recent active long repeats benefited from the PacBio long-reads-based assembly.

A total of 47,526 coding genes were identified in PeaZW6 (Supplementary Tables 9 and 10). The average length of genes and coding sequences was 2,563.7 bp and 1,122.3 bp, respectively (Supplementary Table 9 and Supplementary Notes). The number of genes with gaps in the 3 kb flanking coding sequence decreased from 20% in PeaCaméor to 1% in PeaZW6 (Supplementary Fig. 8a), and the number of transcripts per gene increased from 1.29 to 1.42 (Supplementary Fig. 8b), indicating improvements in the completeness of the regulatory region sequences and the annotation of alternative splicing. The protein mode BUSCO completeness of annotated genes was also higher in PeaZW6 (97.77%) than that in PeaCaméor (93.99%) (Supplementary Table 4). The length distribution of protein-coding genes in PeaZW6 was comparable to that in Medicago with only approximately one-eighth genome size of pea (Fig. 2c). Furthermore, genes with a length of more than 2 kb have a similar pattern of expression breadth (Fig. 2d). These results suggest that the high repeat content in the pea genome may have little effect on the gene structure or on the expression profiles of protein-coding genes.

### Genomic polymorphism

**Fig. 3:**
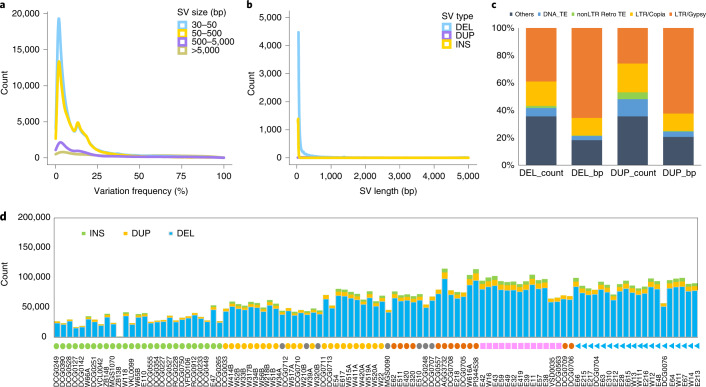
Summary of SVs for 118 representatives cultivated and wild pea in Pisum. **a**, Density plot of variation frequency for different SV size. **b**, Density plot of SV length for different SV type. DNA_TE, DNA transposable elements; LTR, long terminal repeat; nonLTR Retro TE, non-LTR retrotransposable elements. **c**, Distribution of repeat types in SVs of deletions and duplications. **d**, Stacked bar plot of SV number and type for each accession. DEL, deletion; DUP, duplication; INS, insertion.

To investigate genomic polymorphisms in cultivated and wild pea within *Pisum*, a total of 26,250,039 high-quality SNPs and 1,443,829 small indels were identified from a set of 118 *Pisum* accessions after strict filtration (Supplementary Data 2 and Supplementary Notes), of which 64.1% SNPs and 53.0% indels were located in intergenic regions, and only 2.4% SNPs and 1.1% indels were in exons (Supplementary Table 11). A curated set of 376,309 SVs larger than 30 bp was called from 118 *Pisum* accessions and mainly composed of deletions (94.5%) (Supplementary Table 12 and Supplementary Notes). The analyses of the SVs indicated that most SVs were small and were present at a relatively low variation frequency (Fig. 3a,b). In addition, it was found that 85.5% and 77.4% of deletions and duplications, respectively, were from repeat sequences, which were dominated by LTR/Copia and LTR/Gypsy (Fig. 3c). The number of SVs for each accession ranged from 916 to 114,900, with an average of 63,987. Compared to cultivated *P. sativum*, accessions of *P. fulvum* and *P. abyssinicum* had more SVs against the PeaZW6 reference genome (Fig. 3d).

### Population genetic structure

**Fig. 4:**
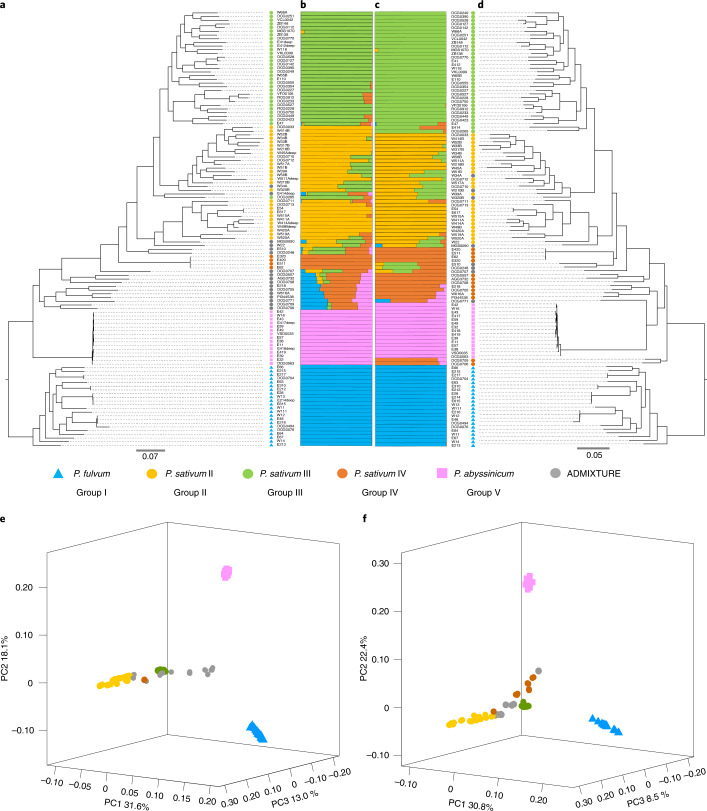
Population genomic analyses of 118 representative cultivated and wild pea in *Pisum* based on SNPs and SVs. **a**, SNP-based phylogenetic tree. **b**, SNP-based ADMIXTURE analysis at *K* = 5. **c**, SV-based ADMIXTURE analysis at *K* = 5. **d**, SV-based phylogenetic tree. **e**, SNP-based principal-component (PC) analysis. **f**, SV-based principal-component analysis. Colors and shapes indicate the genetic groups and taxonomic species in *Pisum* of each accession, respectively.

To clarify the phylogenetic relationship and population genetic structure of cultivated and wild peas within *Pisum*, ADMIXTURE was applied to both SNP and SV datasets, and the results were highly consistent (Fig. 4b,c and Supplementary Fig. 9). The structure with three distinct species in *Pisum*, *P. fulvum*, *P. sativum* and *P. abyssinicum* received unanimous support. Three genetic groups were identified within *P. sativum*, of which *P. sativum* IV (PSIV) represented an earlier differentiated group (Fig. 4b,c). *P. sativum* II (PSII) and *P. sativum* III (PSIII) mainly corresponded to two genetic groups representing cultivated peas in different geographical regions (that is, Asia and Europe), which may be related to the transmission route after pea domestication (Fig. 4b,c). Phylogenetic trees constructed with SNP and SV datasets (Fig. 4a,d) showed similar phylogenetic relationships for the main branches and good correspondence to the major genetic groups of ADMIXTURE results. In addition, *P. fulvum*, *P. abyssinicum* and cultivated *P. sativum* of *Pisum* formed three separate single clades (Fig. 4a,d), which were also supported by principal-component analyses of the SNP and SV datasets (Fig. 4e,f and Supplementary Notes).

### *Pisum* diversity and linkage disequilibrium

Based on the results of ADMIXTURE, genetic diversity was first calculated for each species within *Pisum* and each genetic group of *P. sativum* with SNPs. Among the three species, *P. sativum* showed the highest nucleotide diversity (π = 9.40 × 10^−4^) followed with *P. fulvum* (π = 7.22 × 10^−4^) and *P. abyssinicum* (π = 2.44 × 10^−4^) (Supplementary Fig. 10a). Of the three genetic groups of *P. sativum*, *P. sativum* II retained the largest nucleotide diversity of all (π = 9.13 × 10^−4^); the nucleotide diversity in *P. sativum* III decreased to approximately two-thirds of the total (π = 6.32 × 10^−4^) (Supplementary Fig. 10b).

In addition, population genetic differentiation (*F*_ST_) was estimated among species and genetic groups with SNPs. In general, the interspecific differentiation was greater than the intraspecific differentiation in *Pisum* (Supplementary Fig. 10). Among the three species, genetic differentiation between *P. fulvum* and *P. abyssinicum* was the highest (*F*_ST_ = 0.563), followed by that between *P. abyssinicum* and *P. sativum* (*F*_ST_ = 0.522) and that between *P. fulvum* and *P. sativum* (*F*_ST_ = 0.440) (Supplementary Fig. 10a). Among the three genetic groups, *P. sativum* II and *P. sativum* III showed the lowest genetic differentiation (*F*_ST_ = 0.175) (Supplementary Fig. 10b), which is consistent with the phylogenetic analyses (Fig. 4a,d).

Linkage disequilibrium (LD) (*R*^2^) was calculated with SNPs but varied among species within *Pisum* and different genetic groups of *P. sativum* (Supplementary Fig. 11). The LD dropped to half its maximum value at 6 kb in *P. fulvum*, whereas the LD extent in *P. sativum* was ~25 kb, similar to that in wild soybean (*Glycine soja*, 27 kb)^7^ and wild maize (*Zea mays* ssp. *parviglumis*, 22 kb)^39^. In *P. sativum* II and *P. sativum* III, the LD decay distance was increased, to 80 kb and 35 kb, respectively.

### Selective signals during pea domestication

To identify putative selective genome regions that were putatively selected during pea domestication, the cross-population composite likelihood ratio test (XP-CLR)^40^ was performed with different comparisons of *P. fulvum* versus *P. sativum* and *P. fulvum* versus *P. abyssinicum*. Between *P. fulvum* and *P. sativum*, 514 sweeps encompassing 7,279 genes covering 15.54% (~578 Mb) of the assembled genome were identified (Supplementary Data 3 and Supplementary Fig. 12a,c,e). Between *P. fulvum* and *P. abyssinicum*, 609 sweeps containing 10,132 genes comprising 19.34% (~719 Mb) of the assembled genome were detected (Supplementary Data 4, Supplementary Fig. 12b,d,f), The candidate selected regions contain several genes homologous to genes related to pod dehiscence and seed dormancy in *G. max* and *M. truncatula* (Supplementary Data 5). An analysis of the genes within the putative selected regions indicated that 1,494 genes were found to be common to *P. sativum* and *P. abyssinicum*, whereas 5,785 and 8,638 genes were unique to each, respectively. Gene Ontology (GO) analysis of 8,638 candidate selected genes unique to *P. abyssinicum* revealed enrichment of genes involved in responses to abiotic and biotic stimuli (Supplementary Table 13).

### QTL analysis and rediscovery of Mendel’s genetic loci

**Fig. 5:**
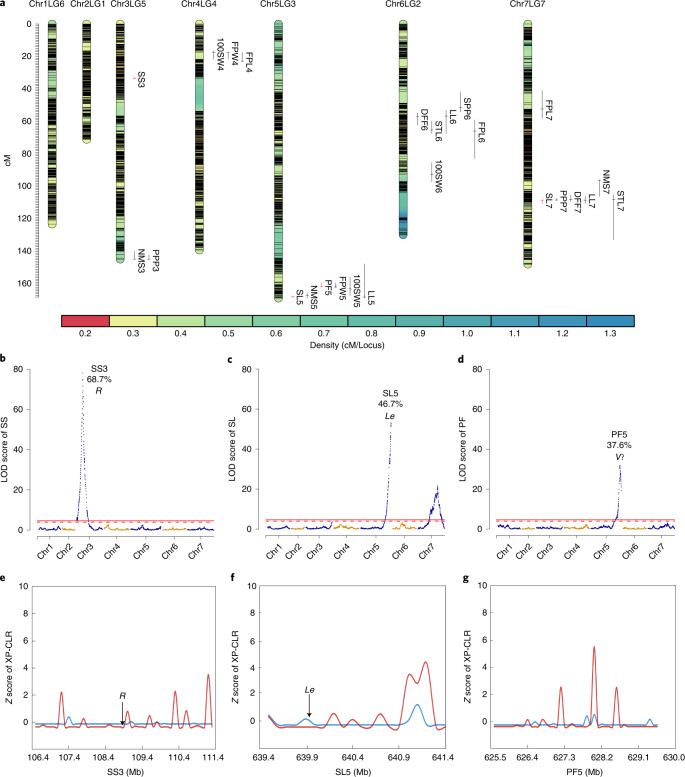
Results of QTL analysis for 12 agronomic traits in pea as well as candidate gene and selective signals in three QTLs associated with three Mendel’s traits. **a** , 25 QTLs were identified to be associated with 12 agronomic traits, and red bars indicate four QTLs in related to the three Mendel’s traits of seed shape (SS), stem length (SL) and pod form (PF). **b**–**d**, distribution of LOD score, PVE and candidate genes in SS3 (**b**), SL5 (**c**) and PF5 (**d**), with the red solid and broken lines representing thresholds of 0.01 and 0.05, respectively. **e**–**g**, Candidate selective signals in SS3 (**e**), SL5 (**f**) and PF5 (**g**) based on results of XP-CLR analysis between species within *Pisum*, with the red line representing *P. fulvum* versus *P. sativum* with *α*_0.05_ = 2.18 and the blue line representing *P. fulvum* versus *P. abyssinicum* with *α*_0.05_ = 0.39.

To explore the genetic basis of important agronomic traits in pea, QTL analysis was performed for 12 agronomic traits in a 300 F_2_ population (WJ×ZW6) using genotyping-by-sequencing (Supplementary Data 6 and 7, Supplementary Fig. 13 and Supplementary Notes). A total of 124,900 high-quality SNP markers were clustered into 2,950 bin markers, and a high-density (0.31 cM) genetic linkage map assembled into seven linkage groups spanning 924.1 cM was constructed (Supplementary Table 14 and Supplementary Fig. 14). Twenty-five QTLs were found to be associated with the 12 agronomic traits, with logarithm of odds (LOD) values ranging from 4.2 to 78.1 and the largest phenotypic variation explained (PVE) up to 68.7% (Fig. 5a and Supplementary Data 8). Of the 25 QTLs, SS3, SL5 and PF5 related to three traits analyzed by Mendel showed higher LOD (78.1, 53.1 and 31.9) and PVE (68.7%, 46.7% and 37.6%), with sharp QTL peaks in genome (4.87 Mb, 1.85 Mb and 4.43 Mb) (Fig. 5b–d and Supplementary Data 8). The results of homology alignment and functional annotation in SS3, SL5 and PF5 discovered two genetic loci previously known to underlie Mendel’s traits, *R*^41^ and *Le*^42^ (Supplementary Data 9 and 10), and one possible candidate gene (*Psat05G0794700*) associated with pod form (Supplementary Data 11 and 12). However, none of these genes fall in the putative selected regions, implying that they may not be closely associated with pea domestication (Fig. 5e–g).

### Pan-genome based on 118 cultivated and wild pea

For a deeper understanding of the *Pisum* diversity, a pan-genome analysis was performed based on individual de novo assembly of 118 cultivated and wild pea accessions (Supplementary Data 13). By aligning individual assemblies to the PeaZW6 reference, we found that the percentages of novel sequences and genes were similar within a genetic group but increased as the group’s genetic distance to ZW6 increased (Supplementary Data 14 and Supplementary Notes). Meanwhile, after merging the new sequences to remove redundancies beyond PeaZW6, we also found that the percentage of new sequences from all accessions was higher than any genetic group (Supplementary Data 15), which indicated that a large portion of diversity of *Pisum* was mainly among different groups in the form of uniqueness of genomic sequences.

**Fig. 6:**
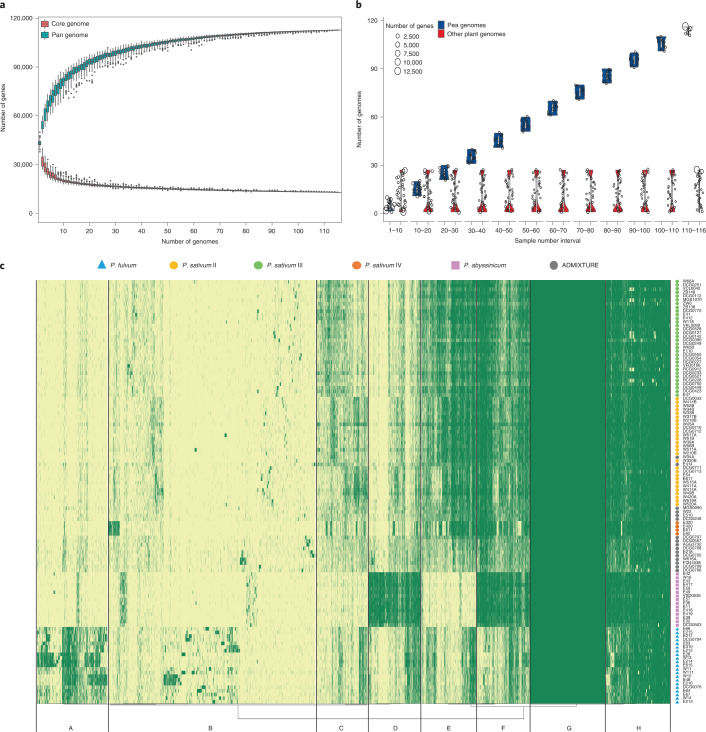
A pan-genome based on 116 representatives cultivated and wild pea in *Pisum* (including ZW6 and excluding three accessions). **a**, Modeling of core genome (red curve) and pan genome (blue curve). **b**, Number of genes present in 116 pea genomes (blue) and 27 representative sequenced plant genomes (red). The size of circle represents the number of genes, and the width of the violin plot represents the frequency of genes. **c**, Presence (green) and absence (light yellow) variation pattern of pan-genome orthologues and A–H = eight clusters according to preferred orthogroups in all accessions. Colors and shapes indicate the genetic groups and taxonomic species in *Pisum* of each accession, respectively.

To further investigate the new sequences related to traits or functions, an analysis of the PAV patterns of *Pisum* pan-genes was conducted (Supplementary Notes). As new genomes added the number of core-genes decreased while the number of pan-genes increased, which gradually converged to saturation (Fig. 6a). After quality control, genes from PeaZW6 and 115 qualified genomes were clustered into 112,776 pan-gene representing phylogenetic hierarchical orthogroups (HOGs), based on the phylogeny of cross-genome orthologues (Fig. 6 and Supplementary Data 16). In *Pisum*, the numbers of core genes, soft-core genes, shell genes and cloud genes were 15,470, 6,170, 41,028 and 50,108, representing 35.19%, 15.54%, 44.28% and 4.99% of the total number of preclustering genes(Supplementary Data 16). The percentage of core genes within any group was higher than the *Pisum* overall (Supplementary Data 16), which was consistent with percentage of novel sequences. Notably, the core percentages of groups were likely corresponding to their calculated genetic diversity (Supplementary Fig. 10), which suggested that the genetic diversity could have also contributed to the percentage of core genes. Meanwhile, the core genes also tended to be more conserved among 27 other plant genomes (Fig. 6b and Supplementary Data 17), suggesting their roles of fundamental functions. Moreover, the neighbor-joining tree of PAVs also showed clear separation of 116 *Pisum* accessions, which is highly consistent with the results based on SNPs and SVs (Supplementary Fig. 15), suggesting the important genetic variations contributed to domestication of *Pisum* were also buried in PAVs.

To inspect the gene preference and functional enrichment in the pan-genome, HOGs were further clustered by PAV patterns into eight clusters named A to H (Fig. 6c and Supplementary Notes). The pattern showed that the *P. fulvum* and *P. abyssinicum* accessions were abundant in unique genes, indicating their potential value as future breeding resources. Many *P. sativum* accessions showed gene intersections with other groups, which might reflect potential events of gene penetration in their breeding history.

Finally, the GO functional enrichment of PAV clusters, genetic groups and unique pan-genes in genetic groups showed diverged functional enrichment between conserved genes (core and soft-core genes) and variable genes (shell and cloud genes). The conserved genes were enriched in fundamental functions such as carbohydrate and lipid metabolic processes. The variable genes were enriched in accessory functions such as stress and stimulus response. Notably, the unique pan-genes of *P. abyssinicum* were enriched in stimulus and chemical response, whereas the pan-genes of *P. fulvum* were enriched in development, growth, reproduction, cytoskeleton and tropism (Supplementary Fig. 16 and Supplementary Notes). This has further confirmed the potential value of *P. abyssinicum* and *P. fulvum* as breeding materials to improve the resistance and production of pea cultivars in the future.

## Discussion

Pea is one of the most important legume crops with high nutritional value and biological nitrogen fixation capacity^43,44^, which has also been a model plant species for genetic studies since the discovery of Mendel’s laws of inheritance^45^. High-quality reference genomes and annotations provide fundamental resources for characterizing genetic traits in crops. Unfortunately, the crop has lacked a high-quality reference genome and genetic transformation system for a long time, thereby losing its dominance and becoming an orphan crop in the modern genomics era^46–48^. In this study, by generating a novel assembly based on full PacBio SMRT long-read sequencing, the genome has increased 243-fold in contig length, showing remarkable improvements in the continuity and quality of complex repeat regions and transposable elements that remained as gaps in the previous reference genome^34^ (Table 1, Supplementary Figs. 7 and 8, Supplementary Table 8 and Supplementary Notes). The new reference genome broadens our knowledge of the genetics underlying the giant size of the pea genome and will facilitate future breeding studies that may help feed the world.

Despite numerous studies focused on the classification of *Pisum*, this long-standing issue remains unresolved, and much confusion about pea domestication persists^49–53^. One point of contention is the taxonomic status of *P. abyssinicum*, namely, whether to regard it as an independent species or subspecies within *P. sativum*^54^. In view of its unique morphological characteristics, degree of reproductive isolation and specific distribution areas^51,54,55^, as well as the results of phylogenetic analyses using genomic SNPs, SVs and PAV identified in the pan-genome, we strongly support *P. abyssinicum* as an undoubtedly separate species distinct from *P. fulvum* and *P. sativum* within *Pisum* (Figs. 4 and 6, Supplementary Figs. 9 and 15 and Supplementary Notes). In addition, there remains skepticism of the traditional understanding of *P. sativum* subsp. *elatius* as the possible ancestor of the modern pea^56^. A high rate of introgression was observed in *P. sativum* subsp. *elatius* (Fig. 4), implying that it may be the product of hybridization between cultivated and wild pea. This hybrid origin was also supported by a recent admixture analysis of wild *P. sativum* groups including the northern *humile*, southern *humile* and *P. sativum* subsp. *elatius*^57^.

Pod dehiscence and seed dormancy are two key traits during legume domestication^58^. Molecular genetic studies have identified several genes controlling these two traits and evidences of parallel selection across legume species^59,60^. One gene believed to be related to pod dehiscence in pea is *Dpo1*^61,62^, a homologue of peptidoglycan-binding domain protein (PGBD) in *M. truncatula* (*Medtr2g079050*)^58^. Based on homologous alignment, *Dop1* was annotated as *Psat05G0678800* in the PeaZW6 genome and localized in the putative selected region of *P. abyssinicum* but not in that of *P. sativum* (Supplementary Data 5), indicating that it may have undergone independent domestication in the two species, as mentioned in a previous study^55^. *GmHs1-1* and *GmG* were demonstrated to control seed dormancy in soybean^63,64^. Two homologous genes *Psat02G0081200* and *Psat02G0507900* corresponding to *GmHs1-1* and *GmG*, respectively, were identified in PeaZW6, and both were present in the putative selected region of *P. abyssinicum* (Supplementary Data 5 and Supplementary Notes).

Gregor Mendel pioneered genetic research through the study of seven characteristics of pea^29,30^. In past decades, four of Mendel’s genetic loci, those controlling seed shape(*R*/*r*)^41^, stem length (*Le*/*le*)^42^ and cotyledon color (*I*/*i*)^65^, as well as seed coat and flower color (*A*/*a*)^66^, have been functionally analyzed, whereas the gene identity of the three other Mendel’s traits including pod color (*GP*/*gp*), pod form (*V*/*v*) and flower position (*Fa*/*fa*) remain unexplored^29,30^. With the available of the reference genome PeaZW6, the four cloned Mendel’s genes were localized precisely (Fig. 1 and Supplementary Data 12). Interestingly, three genes showed the same mutation alleles as found in previous studies, whereas for the mutation of the *r* gene in ZW6 (*Psat03G0136800*), a 9-bp insertion in exon 22 instead of a 0.8-kb insertion^41^ resulted in a transition phenotype of pitted seeds rather than wrinkled seeds (Supplementary Data 12). Meanwhile, QTL analysis enabled the rediscovery of two Mendel’s genes, *r* and *le*, as well as candidates for the *v* gene in three major QTLs (Fig. 5, Supplementary Data 8–12 and Supplementary Notes).

Several studies have emphasized the need for pan-genomes in order to fully understand the genomic complexity of a species^18,20,21,67^. Individual genomes may contain unique genes that shape unique traits, whereas the core genes shared among many genomes may explain what shapes a species^16,19,22,67^. Due to the technical limitations of NGS, the initial assemblies of 118 accessions were fragmented and incomplete. To overcome this, we introduced a strategy combining two different algorithms based assemblies with reference-guided scaffolding to improve individual assemblies. Empowered by the high-quality PeaZW6 reference, the completeness of de novo assemblies had evidently improved (Supplementary Data 13). We also used a combination of de novo and map-to-pan based strategies for PAV discovery in our pan-genome analysis. This approach enabled us to use NGS resequencing data as much as possible to understand the pan-genome of peas (Supplementary Data 18). The percentages and functional enrichment of core, soft-core shell and cloud genes were consistent with or comparable to those of previous studies^16,21,22,37^, confirming the feasibility of our improved strategy. Overall, the pan-genome analysis revealed the locations of the conserved and diverged parts of the genomes, enhancing our knowledge about the diversity and potential value of different pea genomes. Nevertheless, based on the NGS-only data, the pan-genome analysis was quite limited. For example, the pan-genome length of the graph-based genome is much smaller than that of the merged and augmented genomes (Supplementary Data 15), indicating that many SVs were not identified in the graph. Such limitations could hopefully be improved with more long-read based individual assemblies.

In summary, the high-quality reference genome and pan-genome presented here provide insights into pea genome evolution and domestication as well as valuable genomic resources for pea genetics and breeding research^22,37^. This study will fill the gap between previous basic models and modern genomics to boost research and crop improvement for the pea.

## Methods

### Sampling and genome sequencing

Chinese pea variety Zhongwan 6 (ZW6), G0005527 in National Genebank of China, was purified by single-seed-descend for three generations. The young leaf of ZW6 was used for genomic DNA extraction. A total of 1031.25 Gb NGS data were generated using Illumina NovaSeq 6000 or Illumina HiSeq X Ten Sequencing platform (Illumina). Meanwhile, 379.34 Gb SMRT sequencing data from PacBio Sequel platform (Pacific Biosciences) was used for assembly analysis.

### Genome size estimation

The genome size of ZW6 was estimated through flow cytometry^68^. Samples were placed in a 500 μl Nuclei Extraction buffer, chopped with a sharp blade and then filtered through a 50-μm filter after 60 s. Five thousand cells were collected for each sample followed by adding 2,000 μl staining buffer with RNase for 30 minutes in the dark. The nuclei suspension was analyzed by CyFlow Space Flow Cytometer (Sysmex Partec) and the corresponding FloMax (v2.3) software (Supplementary Fig. 2). The K-mer method was performed using JellyFish (v2.3.0)^69^ (*K* = 21) with ~800 Gb Illumina sequencing data (~187×) to obtain the frequency distribution of distinct K-mers. Based on the distribution, GCE (ftp://ftp.genomics.org.cn/pub/gce) was used to estimate genome size, heterozygous ratio and percentage of repetitive sequence (Supplementary Fig. 3).

### 10x Genomics library construction and sequencing

For 10x Genomics sequencing, high-molecular-weight genomic DNA was extracted, indexed and barcoded according to the Genome Reagent Kit Protocol (10x Genomics). Then, the library was prepared and HiSeq 2500 (Illumina) was used to sequence.

### Bionano sequencing

According to Bionano Prep Plant Tissue DNA Isolation Protocol, high-molecular-weight DNA was extracted from seedling leaves. Then, mimicking enzyme digestion and endonuclease DLE1 was chosen to digest. The labeling and staining processes were implemented according to the Bionano Prep Direct Label and Stain (DLS) Protocol. Bionano Saphyr chip (Bionano Genomics) was used for sequencing.

### Hi-C experiment and sequencing

Fresh leaves were fixed with formaldehyde and filtered for nuclei. Extracted chromatin was digested using *Hind*III restriction enzyme (New England Biolab), and then four Hi-C libraries were constructed (Supplementary Notes)^70^. After quality control, the Hi-C libraries were sequenced on an Illumina HiSeq X Ten sequencer.

### RNA-seq and public data collection

Ten seeds of ZW6 were planted in glasshouse under natural conditions of the Changping Experimental Station of the Institute of Crop Sciences, Chinese Academy of Agricultural Sciences (CAAS), Beijing, in 2014. Eight tissues including root, leaf, tendril, stem, flower, flower bud, green pod and immature seed were harvested at flowering and pod setting stage and immediately placed in liquid nitrogen and stored at −80 °C. The total RNA of each tissue sample was extracted using Trizol-based RNA extraction kit (Novogene). Subsequent mRNA extraction and mRNA-seq libraries were conducted using Kapa transcriptome kits and sequenced with Illumina HiSeq 2000 platform. A total of 32.1 Gb paired-end reads were generated for the eight RNA-seq libraries and deposited in NCBI BioProject PRJNA730094. Public RNA-seq data from PRJNA267198, PRJNA517587, PRJNA277074 and PRJNA328997 were also used for transcriptome analyses.

### Genome assembly

The PacBio reads were de novo assembled using Canu (v1.8)^71^. The assembled contigs were corrected using Pilon (v1.23)^72^. Potential duplicated or haploid contigs were purged using PurgeHaplotigs (v1.1.1)^73^. The purged contigs were further scaffolded with 10x Genomics data using ARCS (v1.0.4)^74^ and LINKS (v1.8.6)^75^. The 10x scaffolds were then corrected and elevated to superscaffolds using Bionano Solve package (v3.4_06042019a) with DLE1 labelled optical map. The superscaffolds were then anchored into chromosome level scaffolds using Juicer (v1.5.6)^76^ and 3d-dna pipeline (v180922)^77^ and manually optimized using JuiceBox Assembly Tools (JBAT) (v1.11.08)^78^. The Hi-C scaffolds was evaluated and anchored to chromosomes using ALLMAPS (v1.0)^79^ with genetic markers from a previous study^38^. The chloroplast genome was manually recovered from assembled contigs using BLAST (v2.5.0 + )^80^ and NC_014057.1 from RefSeq as reference. The mitochondrion genome was manually recovered using BLAT (v34)^81^ with all available mitochondrion genes from NCBI as seed to search the assembled contigs for candidates. Other basic sequence manipulation and statistics were completed using SeqKit (v0.15.0)^82^. The PeaZW6 assembly download, browser and basic analysis tools is available at Pea Genome Database (https://www.peagdb.com/). See Supplementary Notes for detailed information.

### Genome assembly assessment

The gene completeness of the ZW6 and Caméor v1a assembly were assessed with Benchmarking Universal Single-copy Orthologs (BUSCO) (v5.0.0)^83^. The K-mer completeness and heterozygosity of the two genomes were evaluated by Merqury (v1.3)^84^. For mapping summary and statistics, the raw NGS reads were mapped using BWA-MEM (v0.7.15)^85^ and the corrected PacBio reads were mapped using Minimap2 (v2.1)^86^. The quality of repetitive genomic regions was assessed using the LTR Assembly Index (LAI)^87^: (1) LTRharvest in GenomeTools (v1.6.0)^88^ and LTR_FINDER (v1.0.7)^89^ were used to de novo predict the candidate LTR-RTs (full-length LTRs retrotransposon) in the two pea assembly sequences, and (2) LTR_retriever (v2.9.0)^90^ was then used to combine and refactor all the candidates to get the final full-length LTR-RTs. LAI was calculated based on the formula: LAI = (intact LTR-RT length/total LTR-RT length) × 100. See Supplementary Notes for detailed information.

### Genome annotation

RepeatModeler and RepeatMasker (v4.1.1; http://repeatmasker.org/) were used to build a ZW6-specific repeat library by identifying repeat families from the PeaZW6 assembly and to mask repetitive sequences in PeaZW6 assembly. The full-length LTR-RT was identified by LTR_FINDER_parallel (v1.0.7)^89,91^.

Protein-coding genes were annotated using a combination of ab initio, homology-based and transcriptome-based prediction. A total of 71 RNA-seq libraries, including 8 from this study and 63 from public databases, were mapped using HISAT2 (v2.1.0)^92^, and transcripts were constructed using StringTie (v1.3.4)^93^. Constructed transcripts were combined using TACO (v0.7.3)^94^. The open reading frames (ORFs) on transcripts were extracted with TransDecoder (v5.5.0)^95^. The complete ORFs from TransDecoder were used as training set for ab initio prediction by BRAKER2 pipeline (v2.1.5)^96^. For homology-based prediction, protein sequences collected from closely related species and published legume genomes were mapped using GenomeThreader (v1.7.1)^97^. The annotation pipeline and toolkit Funannotate (v1.7.4) (https://funannotate.readthedocs.io/en/latest/index.html)^98^ were used to combine different evidences for a preliminary annotation set. A multilevel curation workflow was applied to reduce potential false predictions. Protein domains on preliminary annotated genes were identified by HMMER (v3.3.1)^99^ against PFAM database (v31)^100^ to remove genes with retrotransposon domains. Single-exon genes suggested by ab initio evidence without expression or homologous were removed. Homology-based search was performed by BLASTP (v2.5.0 + )^80^ against UniProtKB/SwissProt^101^, NR and KEGG^102^ databases, and protein from closely related species and published legume genomes, to remove genes without homology. Finally, frameshifted and partial genes were removed using GFFRead in Cufflinks (v0.11.6)^103^. Functional annotation was performed using InterProScan (v5.0)^104^ and eggNOG-mapper (v2.1.6)^105^ to identify their potential functions based on homology. In addition, BLASTP (v2.5.0 + ) was also used to search NR and KEGG databases for annotation rate and other cross checking. The gene length used in statistics was defined as the chromosomal distance between the start and stop codon. For chloroplast and mitochondrion, the ab initio prediction and ORF extraction was done using genetic code 11. See Supplementary Notes for detailed information.

### Gene expression analysis

The raw RNA-seq reads were quality controlled with Trimmomatic(v0.39)^106^ and FastQC (v 0.11.9)^107^. The trimmed reads were mapped to the final chromosome-level PeaZW6 assembly guided by gene annotation model using HISAT2 (v2.1.0)^92^. The expression level for each gene was determined by StringTie (v1.3.4)^93^.

### Comparative genome analysis

To minimize the effects of homologous genes in the detection of the synteny blocks for Medicago/PeaZW6 and Medicago/PeaCaméor, MCScanX^108^ was used to identify the syntenic region of PeaZW6/Medicago and PeaCaméor/Medicago using all-to-all BLASTP results of reciprocal best hit protein pairs against MedtrA17_4.0^109^. Briefly, all proteins in one genome were BLASTP searched against a protein database of another genome, and vice versa. The E value threshold was 1 × 10^−10^. Orthology was identified if two proteins were each other’s best BLASTP hit. As two parameters (‘s’ and ‘m’) in MCScanX were important to the number of detected syntenic blocks and the number of homologous genes within synteny blocks, we ran the MCScanX with different combinations of “s” and “m” and counted the number of syntenic blocks and the genes harbored, respectively. OrthoFinder (v2.5.4)^110^ was used for gene family construction, and the longest protein was selected to represent loci with multiple transcripts.

### Resequencing and identification of SNPs, indels and SVs

Five seeds of 76 accessions representing different taxa of *Pisum*^50^ were planted in glasshouse under natural conditions of the Institute of Crop Sciences, CAAS, Beijing in 2020. Fresh leaves of one plant for each accession were harvested to extract genomic DNA and resequenced using Illumina NovaSeq 6000 sequencing platform (Illumina). A total of 6.2 T 150-bp paired-end Illumina reads were generated with an average coverage of 14.98× per accession (Supplementary Data 2). In addition, published resequencing data for the 42 accessions of *Pisum* used in a previous study were included in the variant calling and population genetic analyses^34^.

Adapters and low-quality sequences of raw reads were removed using Trimmomatic^106^, and clean reads were mapped to the reference genome of ZW6 using BWA-MEM (v0.7.15)^85^. SNP calling was performed using Genome Analysis Toolkit 4 (GATK4, https://gatk.broadinstitute.org) with default parameters. Raw SNPs and indels were first filtered with the GATK recommended variant filtration and then filtered using VCFtools (v0.1.15)^111^ (Supplementary Notes). Variants were annotated using snpEff 4.3t^112^ based on the PeaZW6 genome annotation.

The SVs were identified with Delly (v0.8.3)^113^ using mapping result in BAM format from resequencing data. First, the SV calling was run on each individual from scratch, and then the results were merged into one VCF file as the guiding reference. Second, the SV calling was run again guided by the combined VCF file. Next, the SVs with PASS tag in filtration were retained for further analysis. Finally, SVs from all cultivars were combined with BCFtools (v1.8)^114^ and filtered using VCFtools (v0.1.15)^111^ (Supplementary Notes).

### *Pisum* population genetic analyses

Finally, one SNP dataset of 118 samples was generated for phylogenetic analysis and other population genetic analyses. The phylogenetic tree was constructed using FastTree (v2.1.10)^115^ with GTR model and visualized with FigTree (v1.4.3) (http://tree.bio.ed.ac.uk/software/figtree/). Population genetic structure was investigated using ADMIXTRUE (v1.3.0)^116^ and the cluster number *K* value was set from 1 to 10. The *K* value with the smallest CV error was assumed to be the best clustering, and *q* values of the primary genetic component of each individual less than 60% were excluded from further analyses of genetic diversity, genetic differentiation and selection. A principal-component analysis was performed using PLINK (v1.90b4.6) with default settings^117^. The first three eigenvectors were kept to plot using R (v3.6.0) (https://www.r-project.org/). The same population genetic analyses with SNP datasets were also conducted using SVs including deletions, insertions and duplications, whereas translocations and inversions were excluded due to their potential uncertainty called from short reads of Illumina sequencing technology.

Nucleotide diversity (π) and *F*_ST_ were calculated for each group based on the best clustering result of ADMIXTURE using VCFtools (v0.1.15)^111^ with a 1,000-kb window and a step size of 100 kb.

LD was estimated using PopLDdecay^118^ pipeline with default parameters for different species in *Pisum* and subgroups of *P. sativum* based on the results of population genetic structure with SNP datasets.

### Genome scan for selective signals

We performed a genome scan using an updated Python version of the cross-population composite likelihood ratio approach (XP-CLR)^40^ released on https://github.com/hardingnj/xpclr. Selective signals across the genome during species divergence within *Pisum* were evaluated in two pairs: *P. fulvum* versus *P. abyssinicum* and *P. fulvum* versus *P. sativum*. Genome scanning was done with a sliding window of 1,000 kb and a step size of 100 kb across the whole genome. The maximum number of SNPs assayed in each window was fixed at 600. XP-CLR values were normalized, and regions above the top 5% highest values were considered as selective regions. Furthermore, selective regions with the top 50% of the reduction of diversity (calculated based on π ratios between cultivated and wild population) were considered as candidate selective regions for accuracy. Finally, adjacent selective regions were merged into selective sweeps using bedtools (v2.30.0)^119^. Results of XP-CLR and reduction of diversity were visualized with R packages CMplot (https://github.com/YinLiLin/CMplot).

### Genetic linkage map construction and QTL mapping

A biparental population was developed consisting of 300 F_2_ individuals from a cross between WJ (female) and ZW6 (male) and grown in the greenhouse under natural conditions in Beijing, China in 2017. Eighteen agronomic traits including 15 quantitative traits and 3 qualitative traits were surveyed (Supplementary Data 6 and Supplementary Notes). Correlation analysis was conducted among different traits using SPSS version 16.0.

DNA from the 300 individuals in F_2_ were genotyped through genotyping-by-sequencing by Novogene (Novogene Bioinformatics Institute, Beijing, China). A total of 805.58 Gb 150-bp paired-end Illumina clean reads were generated and mapped to the PeaZW6 reference genome using BWA-MEM (v0.7.15)^85^. SNP calling was performed using GATK 4 (https://gatk.broadinstitute.org) with default parameters. Raw SNPs were first filtered with the GATK recommended variant filtration and then filtered using VCFtools (v0.1.15)^111^. The final VCF file was converted into ABH-format mapping data file using the Perl script run_pipline.pl in Tassel (v 5.2.40)^120^ and screened for suitable markers to construct the genetic linkage map using R/qtl^121^. SNPbinner^122^ was used to calculate breakpoints and construct genotype bins (Supplementary Notes). A genetic linkage map was constructed with the bin markers using the Kosambi map function in R/qtl^121^. QTL analysis was performed using R/qtl with interval mapping method^121^. Significance thresholds (*α* = 0.05 and *α* = 0.01) were estimated via 1,000 permutations^123^ for each trait. A single QTL model followed by multiple QTL model were applied to identify QTLs with LOD values higher than the threshold and to determine the best fit QTL model for each trait. Results of the genetic map and QTL analysis were visualized with R packages LinkageMapView^124^ and CMplot (https://github.com/YinLiLin/CMplot).

### Mapping of identified Mendel genes

Four identified Mendel’s genes were searched from previous reference^29,30^. The protein ID for round seed was CAA56319.1 (ref. ^41^). The protein ID for tall trait was AAC49792.1 (ref. ^42^). The protein IDs for colored versus unpigmented seed coats and flowers were ADO13282.1 and ADO13283.1, respectively^66^. The protein IDs for yellow versus green cotyledons were BAF76351.1 and BAF76352.1, respectively^65^. BLASTP tools with high confidence (1e^−6^) were used to locate four identified Mendel’s gene in the reference PeaZW6.

### *Pisum* pan-genome assembly, annotation and PAV analysis

Each accession was de novo assembled from resequencing data using DBG-based MEGAHIT (v1.2.9)^125^ and OLC-based MaSuRCA (v3.4.0)^126^ independently. The two assemblies were merged using CD-HIT (v4.8.1)^127^ and anchored to the PeaZW6 reference using RagTag (v2.0.1)^128^ similar to the Panoramic pipeline^129^. The qualities of the 118 assemblies were assessed using BUSCO (Supplementary Data 13), to exclude accessions with deficiency (*C* < 90%) in BUSCO completeness.

Contigs from each individual assembly were aligned to PeaZW6 reference using MUMmer (v4.0)^130^. The aligned segments (identity ≥90%, length ≥100 bp) of contigs were trimmed out. The retained sequences were considered additional to the PeaZW6 genome (Supplementary Data 14). To remove interassembly redundancies, an vg and minigraph-like “augmentation” strategy was used. Starting with the PeaZW6 reference, we iteratively aligned each genome and added additional sequences to the previous augmented reference as new reference for the next round. Meanwhile, the graph-based pan-genomes were also generated from all assemblies using minigraph (v0.13)^131^ with the parameter -l 500 -d 500, and statistics were reported by gfatools(v0.5)^131^. This workflow was also repeated for all genetic groups (Supplementary Data 15).

After soft-masking repeat sequences using RepeatMasker, the BRAKER2 pipeline^96^ was used to predict genes on each genome using PeaZW6 model and protein sequences from PeaZW6, PeaCaméor and SwissProt database as hints. Predicted protein sequences were clustered using CD-HIT (v4.8.1)^127^ to remove duplicated genes. Genes overlapping the repeat elements (≥50% length) were removed. Also, genes were aligned to the PFAM database using HMMER (v3.3.1)^99^ and the UniRef90 database using BLASTP (v2.5.0 + ) to filter out fragmented genes whose length coverage of target sequences was <50%. Finally, the retained genes were aligned to PeaZW6 genes to determine if they are additional genes using BLASTP (v2.5.0 + ) (Supplementary Data 14).

Proteins from all accessions were clustered using OrthoFinder (v2.5.4)^110^ (−*y* enabled for splitting the paralog genes into distinct HOGs) into phylogenetic HOGs as representative of pan-genes. We further used a “map-to-pan” strategy to recover falsely missed HOGs in each accession due to sequencing bias or partial gene predictions. Using complete gene sequences from all accessions as reference, the raw reads from all 116 accessions were mapped using Minimap2 (v2.1)^86^ and limit NM ≤ 1 using samtools^132^. Genes ≥99% length and ≥3× depth covered were considered present in a accession, and their corresponding HOGs were marked as present in the PAV table.

After determining the PAV pattern across 116 genomes from HOGs and map-to-pan (Supplementary Data 18), the final PAV pattern was clustered by the ward.D method in the hclust package and illustrated by the pheatmap package in R (v3.6.0). Based on their percentage of genomes shared, the HOGs were classified into core genes (≥99% of genomes), soft-core genes (≥90% and <99%), shell genes (≥15% and <90%) and cloud genes (<15%), per definitions in Roary^133^, for all accessions and genetic groups. The unique core genes and unique pan-genes for each group were determined by removing genes shared between at least two groups.

To investigate the function of the pan-genes, the clustered pan-genes were cut into eight groups labeled A to H using cutree in R (Fig. 6c). Due to the limitation of 65,535 columns in the hclust package, the randomForest package was used to build a classifier and reassign the 112,776 HOGs into eight prebuilt groups, and the average area under the curve achieved 0.98 in 100 runs (Supplementary Fig. 17). The putative functional enrichment for all groups was assessed using EggNOG-mapper (v2.1.6)^105^ based on EggNOG database (v5.0)^134^. The GO enrichment analysis was carried out using AgriGO (v2.0)^135^ and TBtools^136^ and illustrated using the pheatmap package in R (v3.6.0).

See Supplementary Notes for detailed information.

### Statistics analysis

In GO enrichment analysis, one-sided Fisher’s exact test was applied, and *P* values were adjusted using the Benjamini–Hochberg method^137^.

### Reporting summary

Further information on research design is available in the Nature Research Reporting Summary linked to this article.

## Online content

Any methods, additional references, Nature Research reporting summaries, source data, extended data, supplementary information, acknowledgements, peer review information; details of author contributions and competing interests; and statements of data and code availability are available at 10.1038/s41588-022-01172-2.

## Supplementary information

Supplementary Information Supplementary Figs. 1–17, Tables 1–14, Notes and References.

Reporting Summary

Supplementary Tables Supplementary Tables 1–18.

## Data Availability

Data from the Whole Genome Shotgun project of *Pisum sativum* cultivar Zhongwan6 (PeaZW6) have been deposited at DDBJ/ENA/GenBank under accession JAMSHJ000000000. All raw sequencing data and the 118 pan-genome assemblies have been deposited at NCBI under the BioProject PRJNA730094. The PeaZW6 assembly (10.5281/zenodo.6622409), and the 118 pan-genome assemblies (10.5281/zenodo.6622578) are also available as Zenodo datasets. The PeaZW6 assembly along with genome browser and basic analysis tools are also available at Pea Genome Database (https://www.peagdb.com/).

## Code availability

The custom scripts used in PeaZW6 genome and pan-genome project have been deposited in Zenodo^138^ (10.5281/zenodo.6614849).
