# Supplementary material for: Improved pea reference genome and pan-genome highlight genomic features and evolutionary characteristics
Source: Nat Genet. 2022 Sep 22;54(10):1553–63. doi: 10.1038/s41588-022-01172-2 (PMC9534762; doi:10.1038/s41588-022-01172-2)
Supplement: Supplementary file 1 — Supplementary Figs. 1–17, Tables 1–14, Notes and References. [file 41588_2022_1172_MOESM1_ESM.pdf]

nature portfolio

https://doi.org/10.1038/s41588-022-01172-2

Supplementary information

Improved pea reference genome and pan-genome highlight genomic features and evolutionary characteristics

In the format provided by the authors and unedited

Improved pea reference genome and pan-genome highlight genomic features and evolutionary characteristics

Supplementary Figures

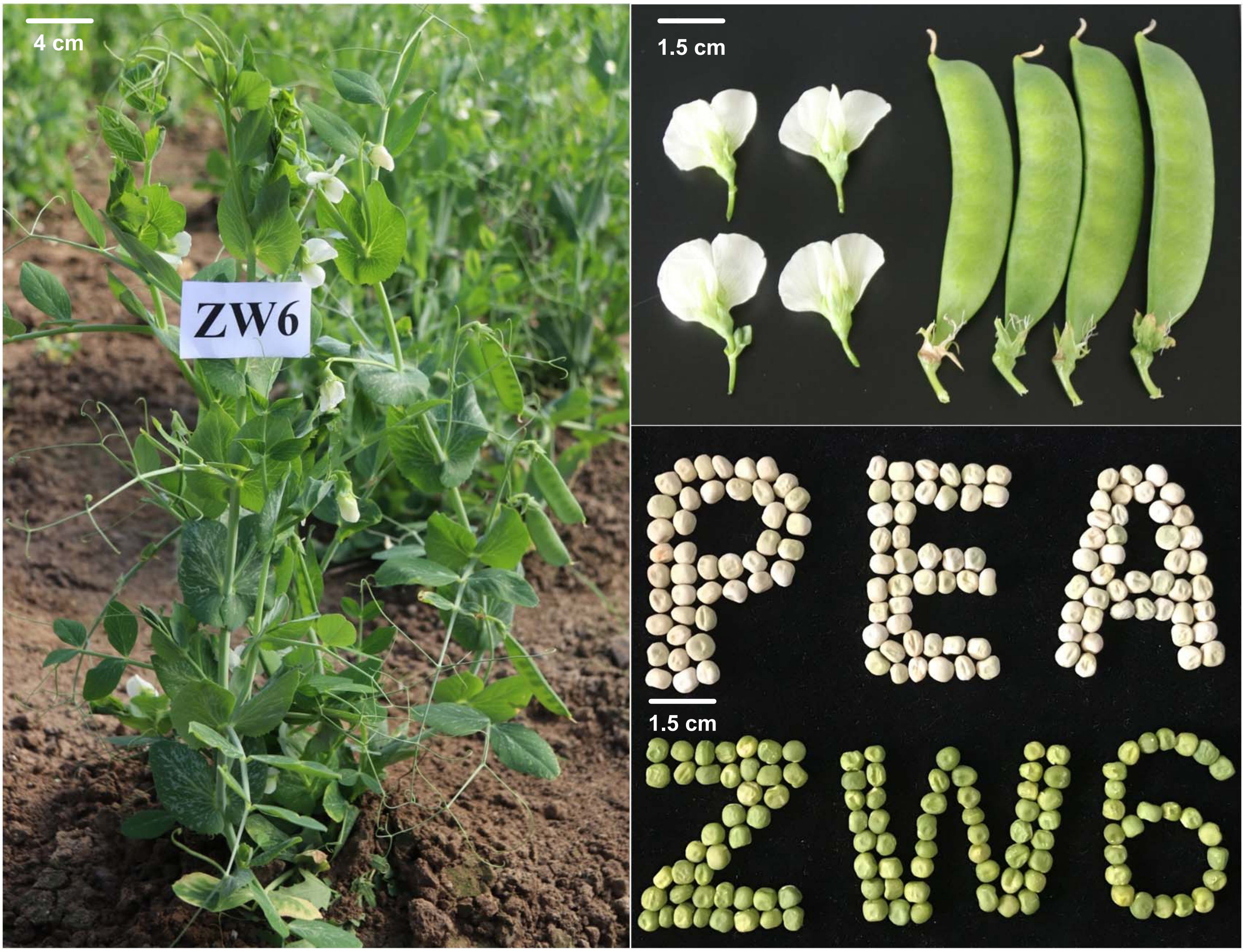

Supplementary Figure 1 ZW6, a Chinese widely grown pea cultivar with unique genetic background used for genome sequencing in this study. Scale bars: 4 cm (left), 1.5 cm (upper right), and 1.5 cm (lower right), respectively.

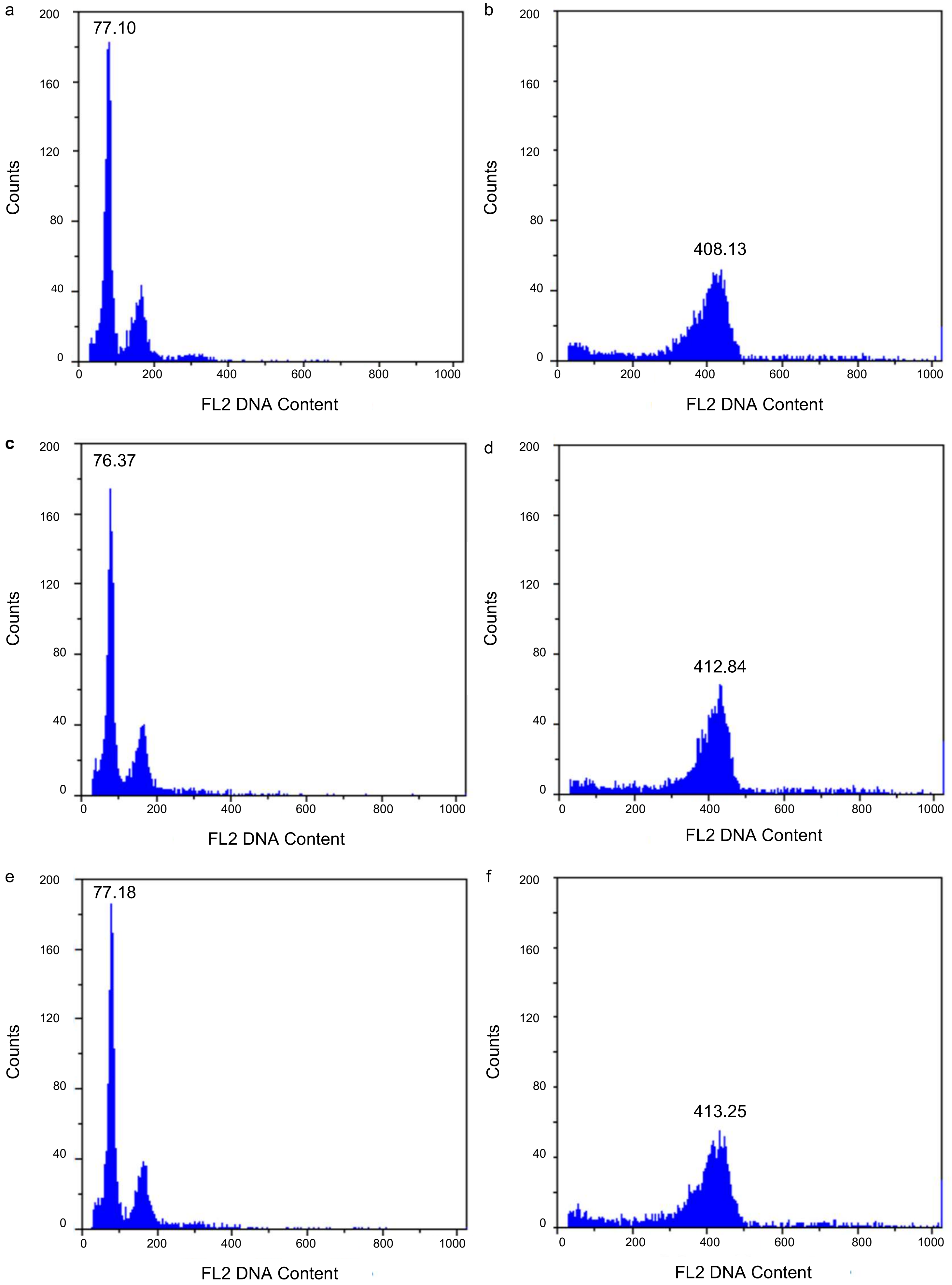

Supplementary Figure 2 Genome size estimation by flow cytometry with three distinct sample repetitions for the standard sample tomato (a, c, e), and the Chinese pea cultivar ZW6 (b, d, f).

K-mer	K-mer number	K-mer depth	Genome size (Mb)	Heterozygous ratio (%)	Repeat (%)
21	864,438,000,024	202	4,261.2	0.08	83.00

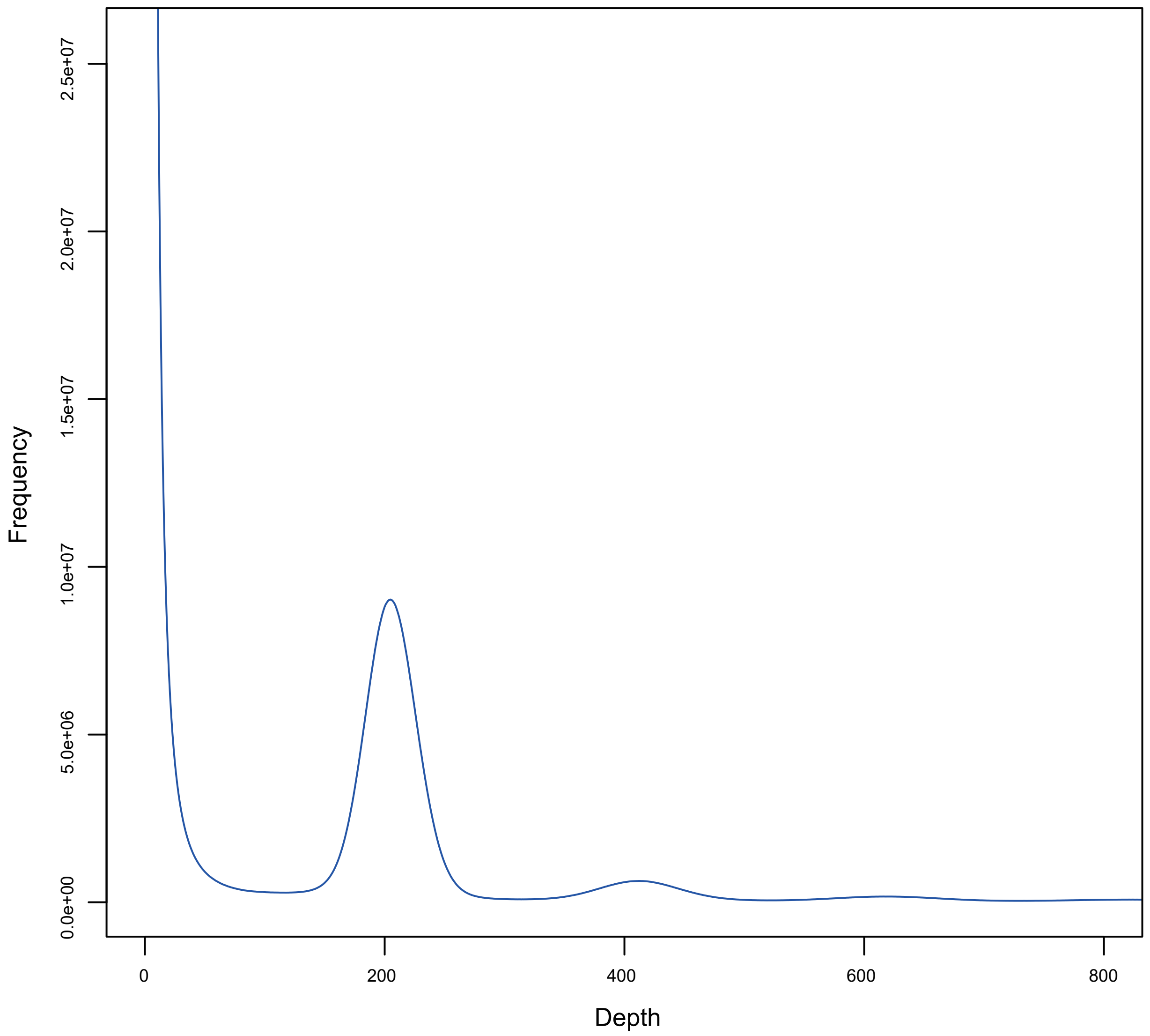

Supplementary Figure 3 Depth distribution of 21-mer in the genome of PeaZW6 by K-mer analysis.

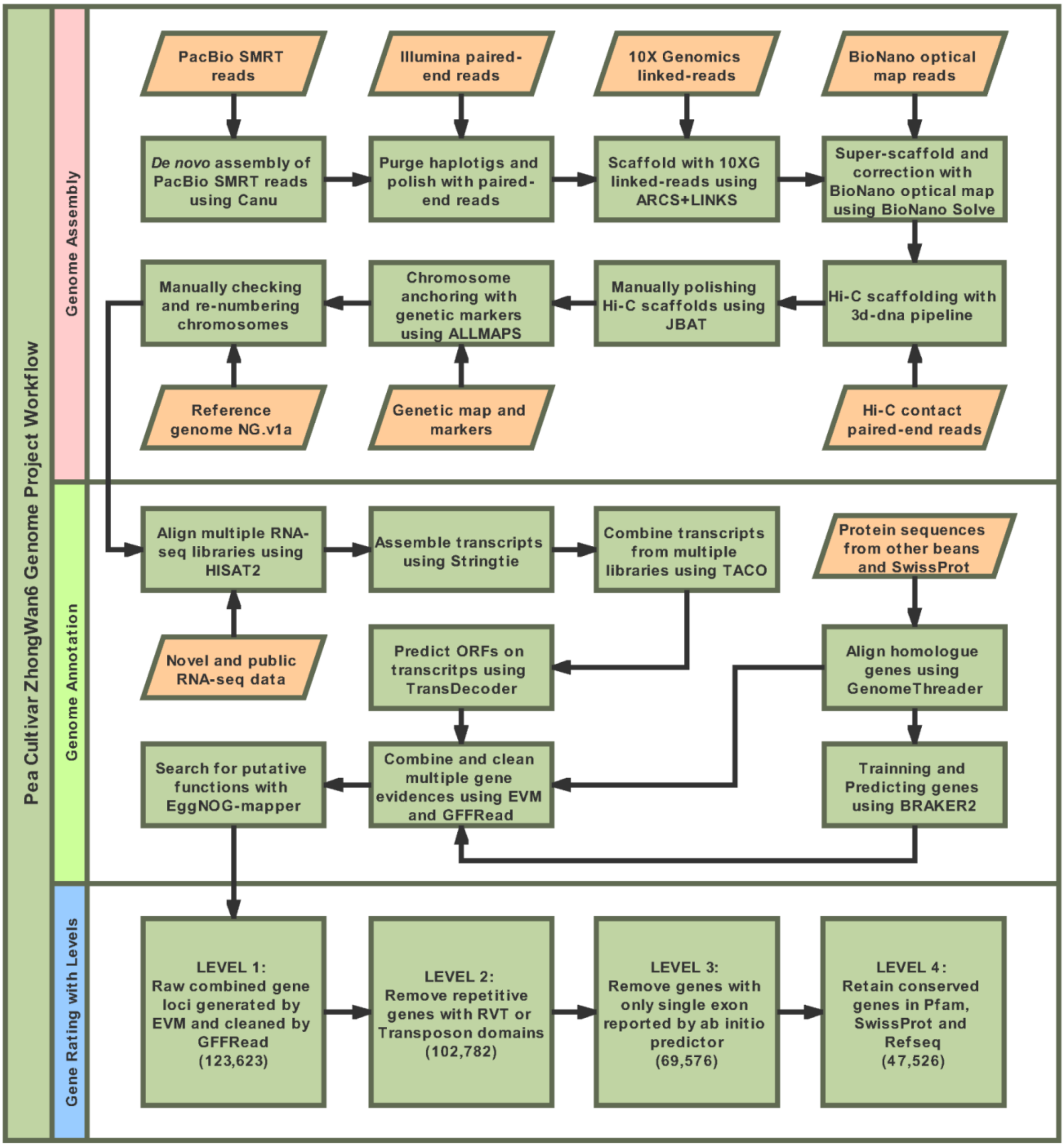

Supplementary Figure 4 Genome assembly and annotation workflow in PeaZW6.

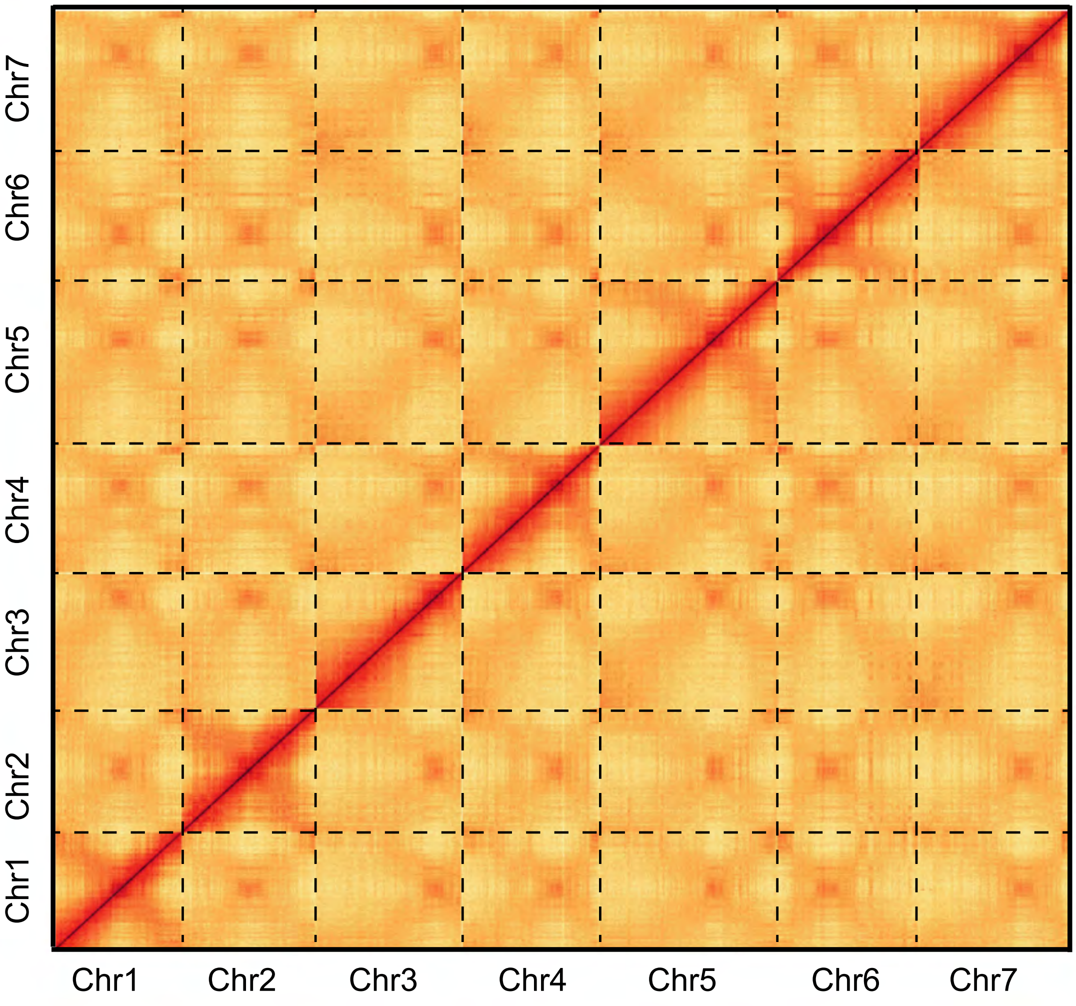

Supplementary Figure 5 Hi-C interaction map of PeaZW6.

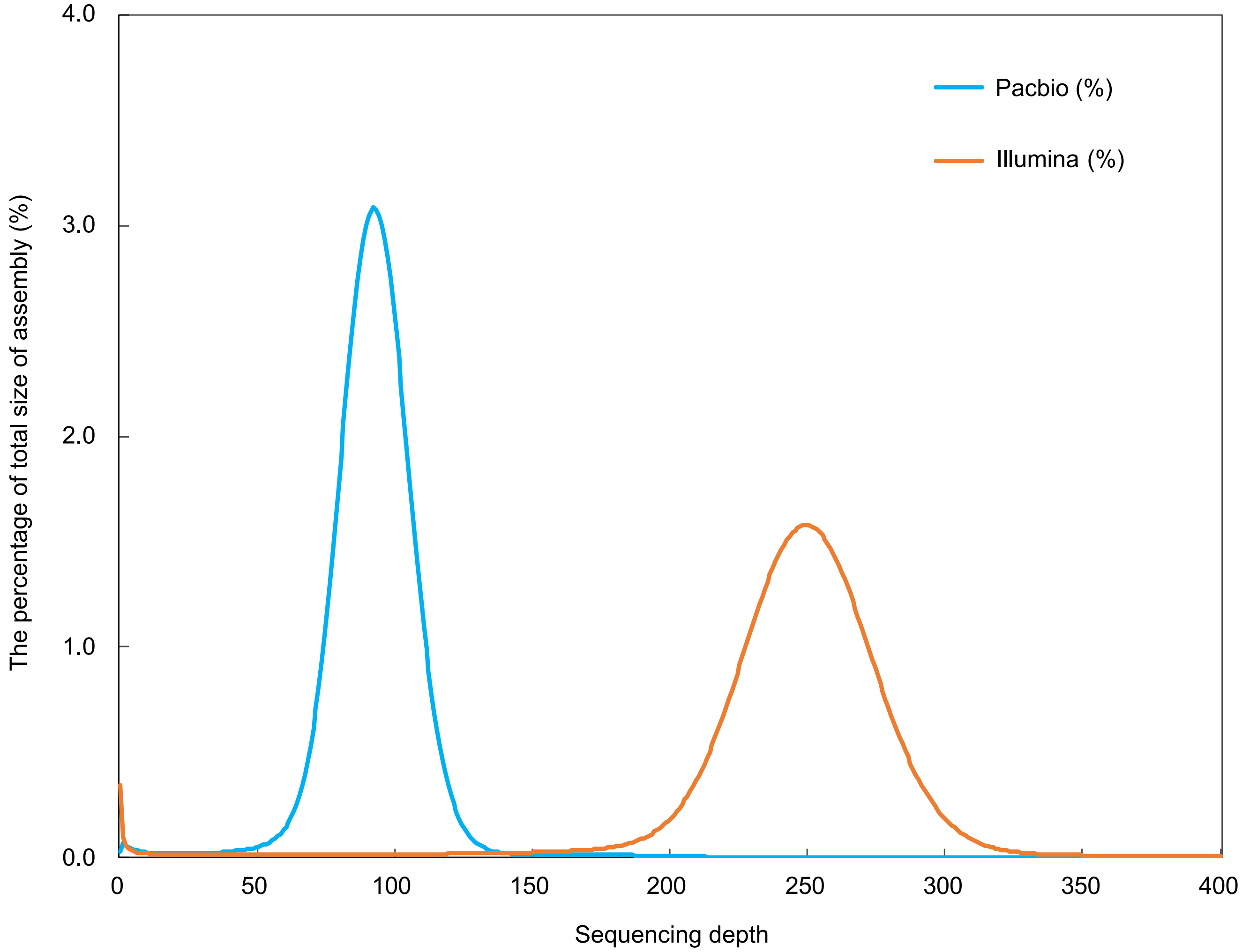

Supplementary Figure 6 The Pacbio (blue) and Illumina (orange) sequences coverage distribution of genome.

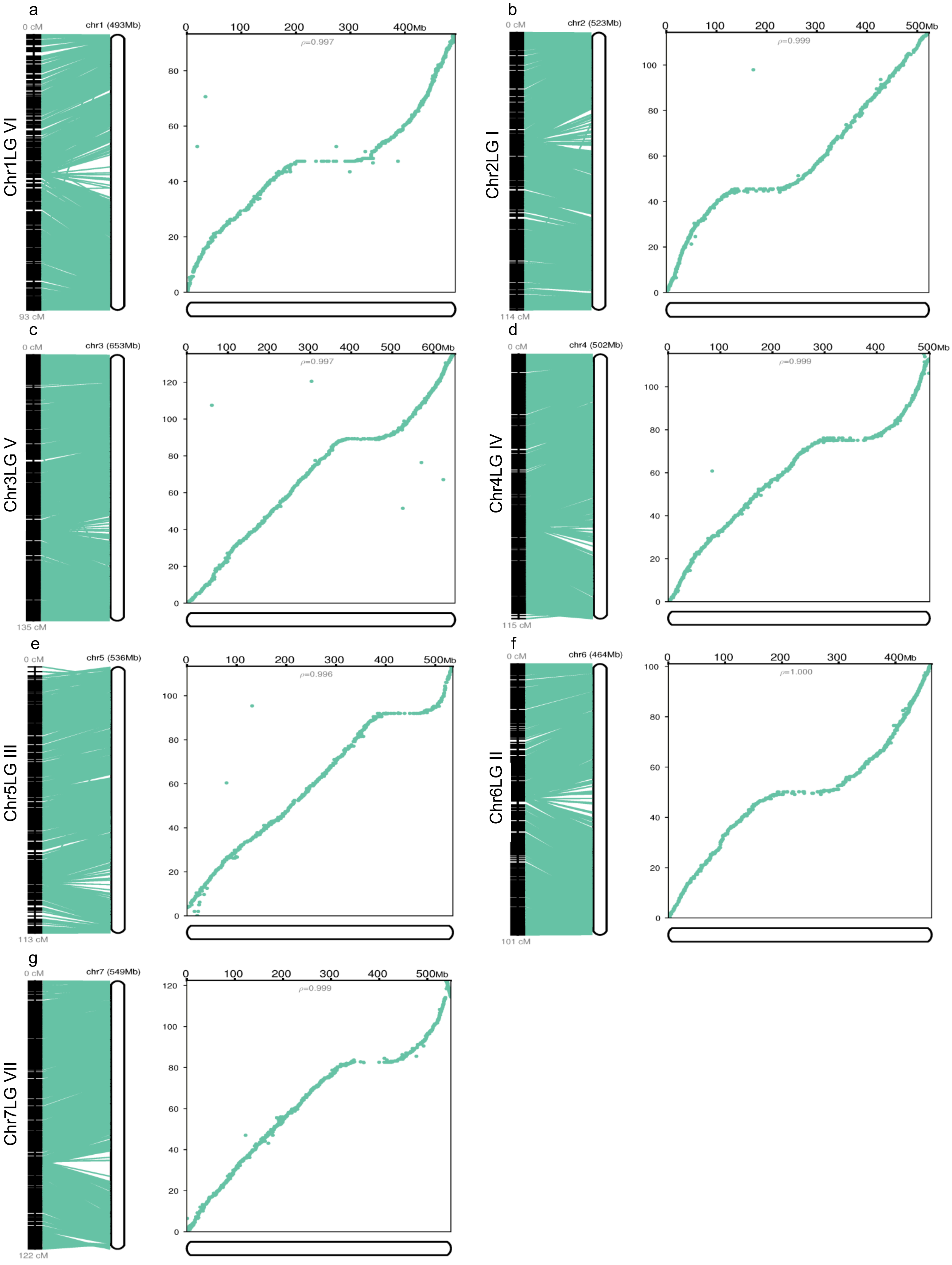

Supplementary Figure 7 High consistency between the assembly of the PeaZW6 and the previous published genetic map[10]. a = Chr1 vs LG VI; b = Chr2 vs LG I; c = Chr3 vs LG V; d = Chr4 vs LG IV; e = Chr5 vs LG III; f = Chr6 vs LG II; g = Chr7 vs LG VII.

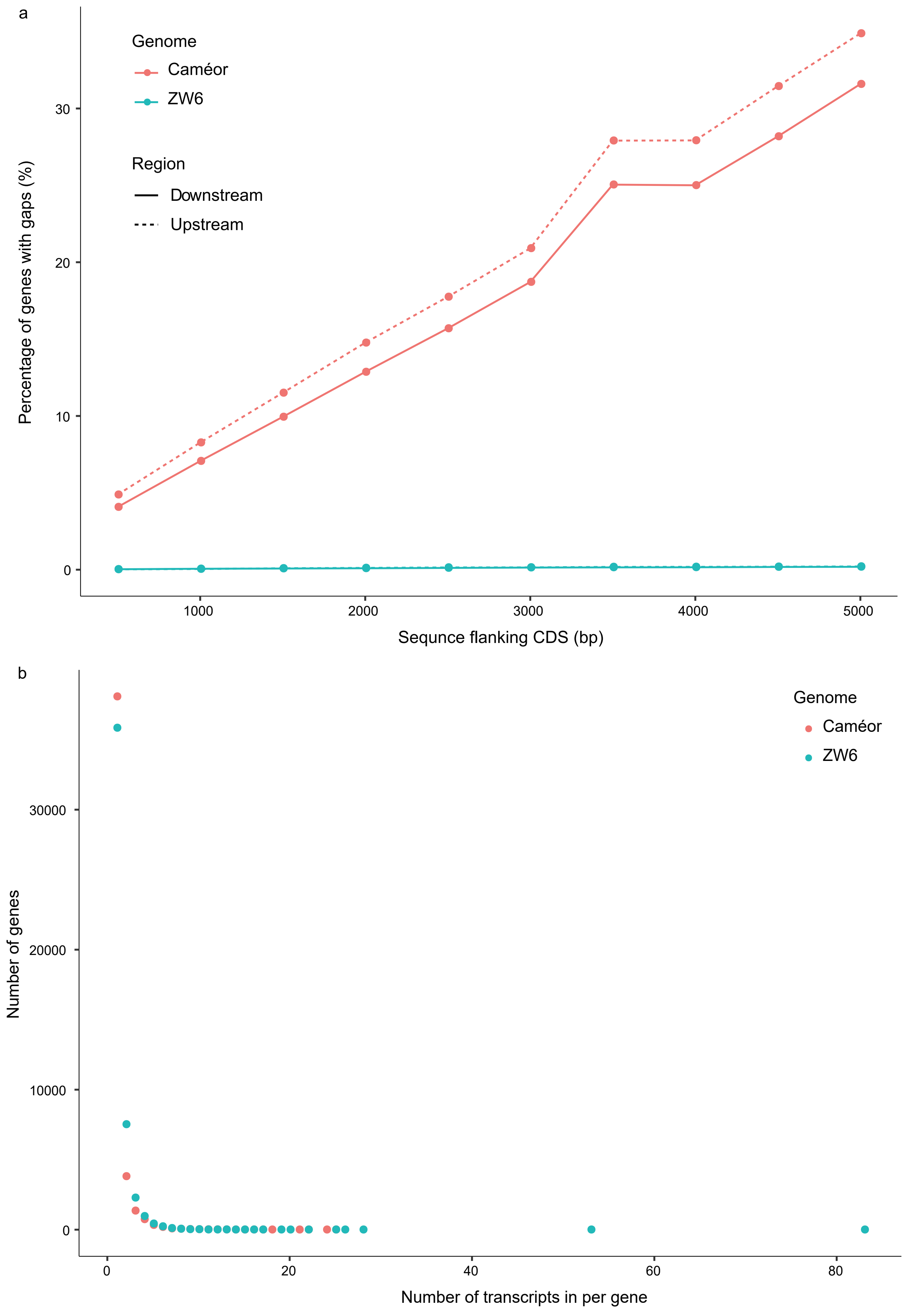

Supplementary Figure 8 Improvement of completeness of regulatory region and the annotation of alternative splicing of PeaZW6 comparing to PeaCaméor. a = Percentage of genes with gaps in CDS flanking regions and b = Number of transcripts in per gene.

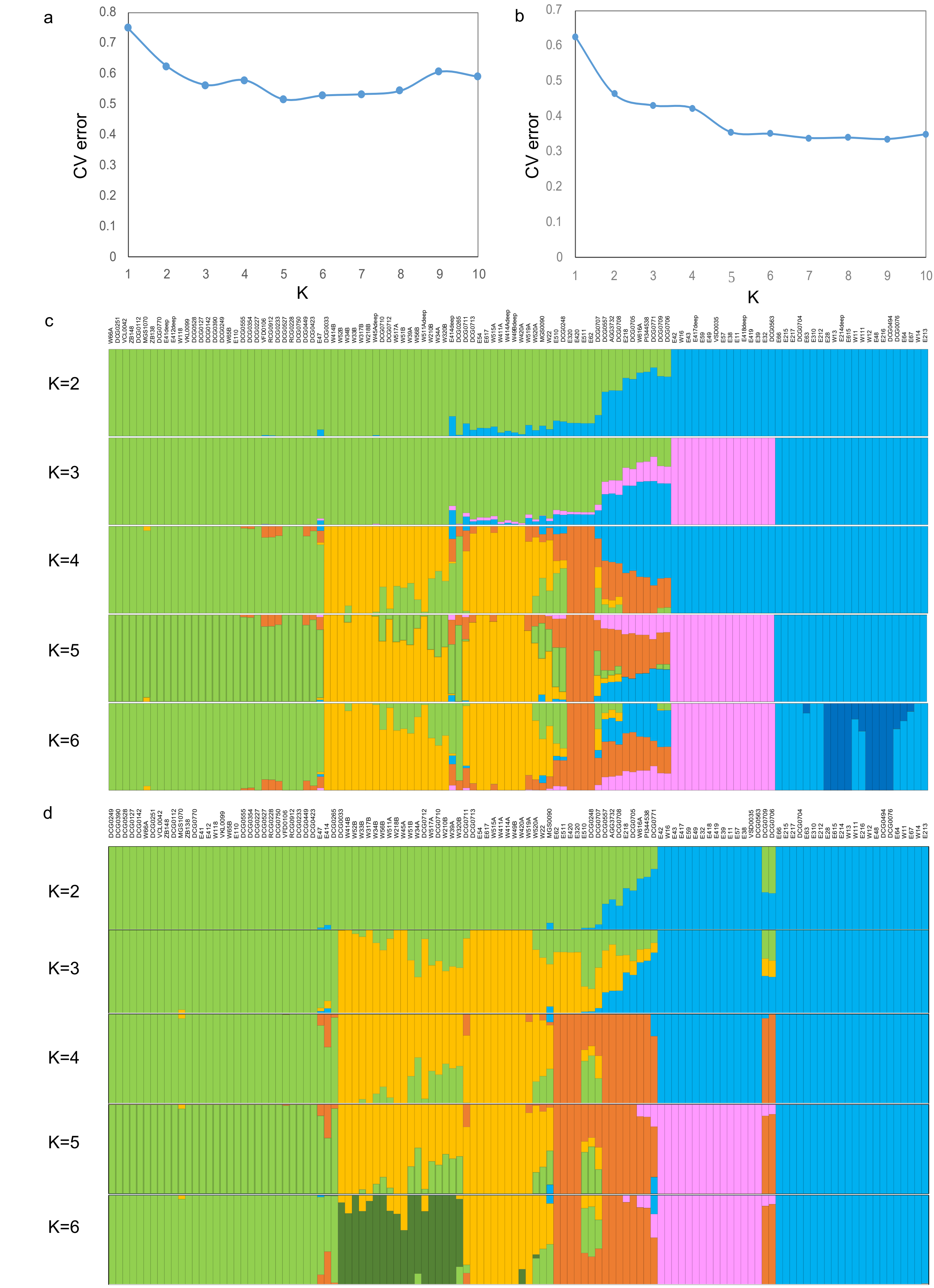

Supplementary Figure 9 CV error values with K ranging from 1 to 10 and population structure inference with K ranging from 2 to 6 based on SNPs (a and c) and SVs (b and d) of 118 accessions of *Pisum*. The accessions are arranged in the same order for the SNP and SV phylogenetic trees, respectively.

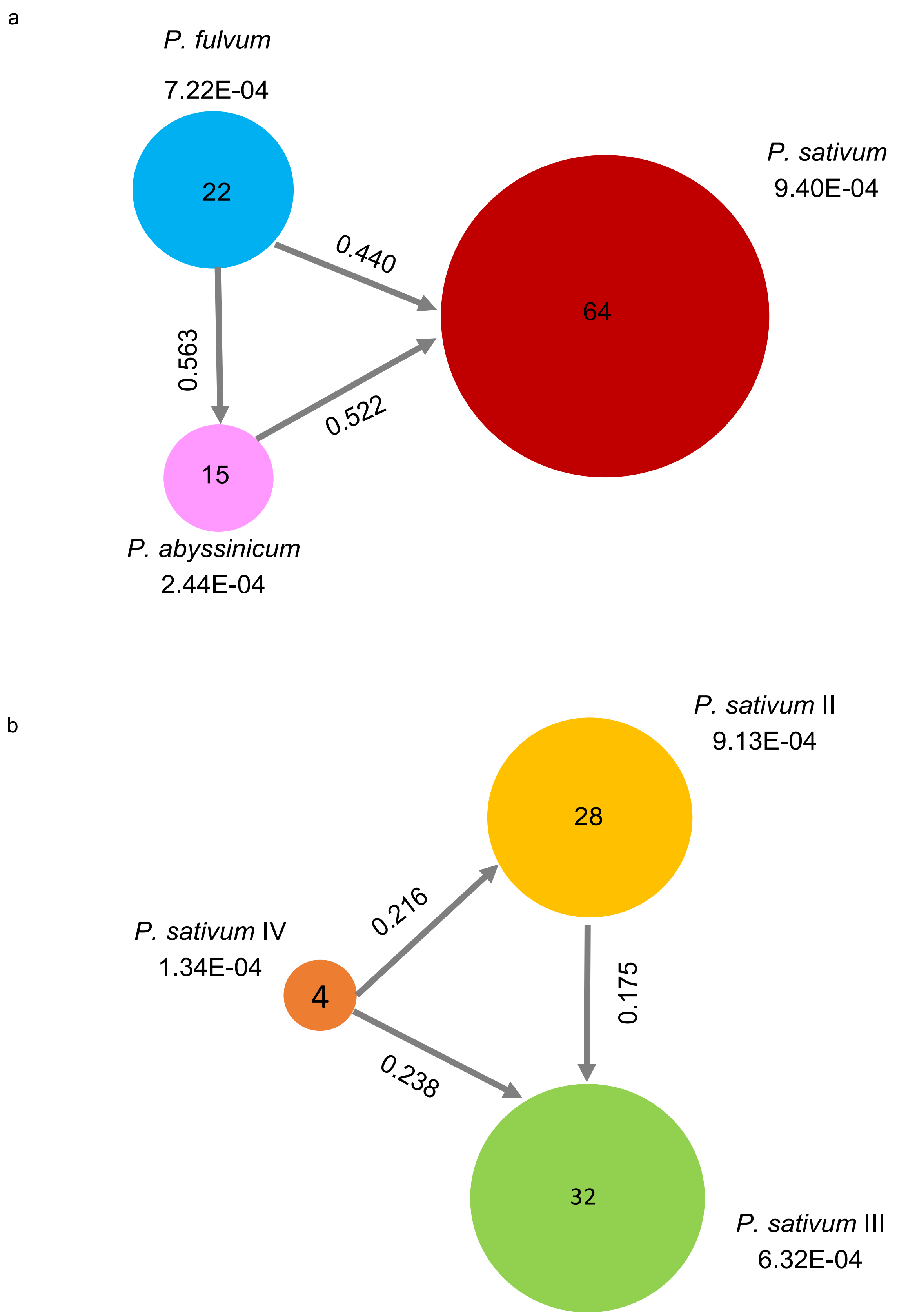

Supplementary Figure 10 Genetic diversity and population genetic differentiation between species within *Pisum* (a) and between subgroups of *P. sativum* (b). The inner numbers and size of circles represent the sample number of species and subgroups. The outer numbers indicate the nucleotide diversity (π) for each species and subgroup, and the values between species and subgroups indicate the population genetic differentiation (F_{ST}) .

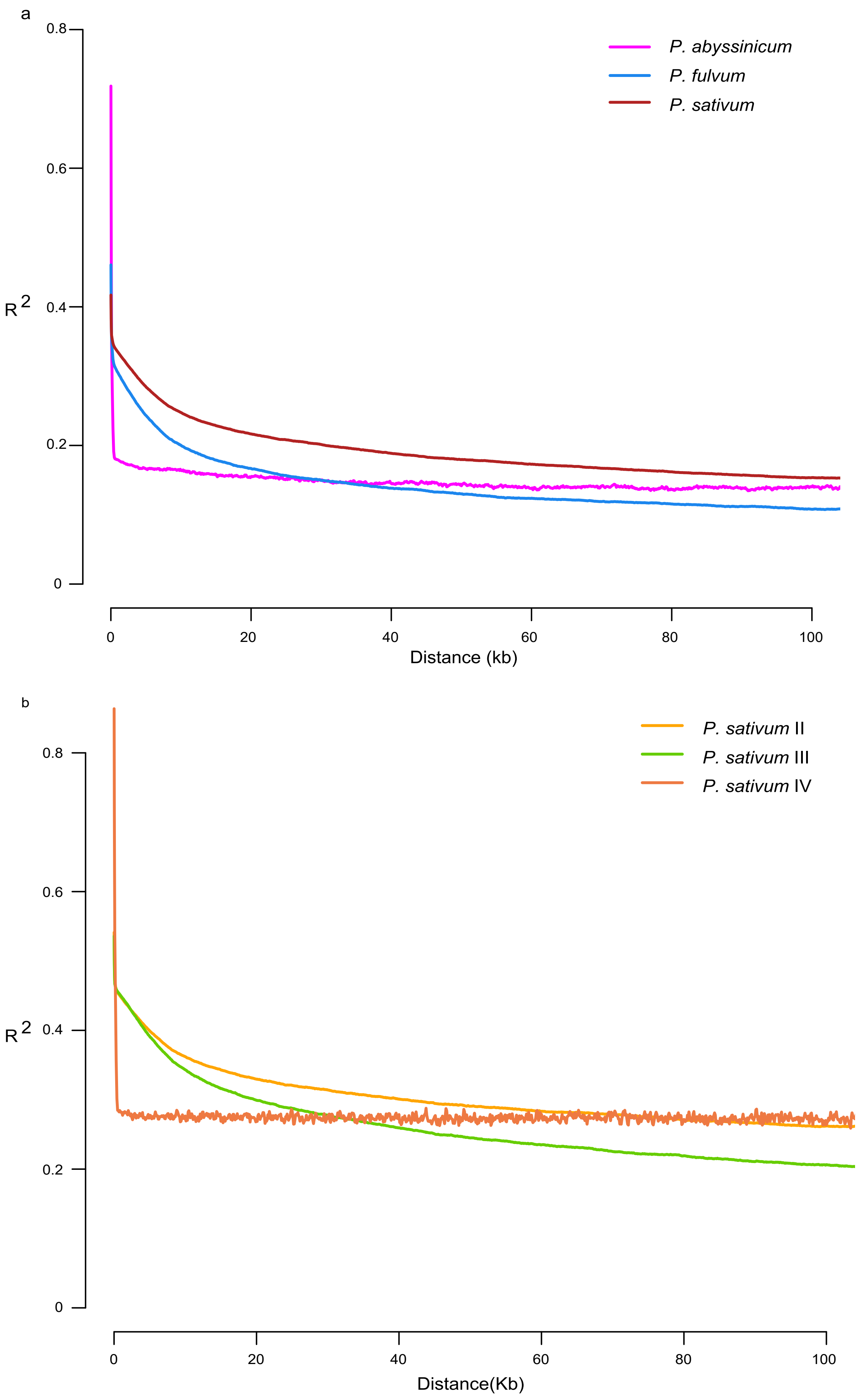

Supplementary Figure 11 Decay of linkage disequilibrium in each species within *Pisum* (a) and each subgroups of *P. sativum* (b).

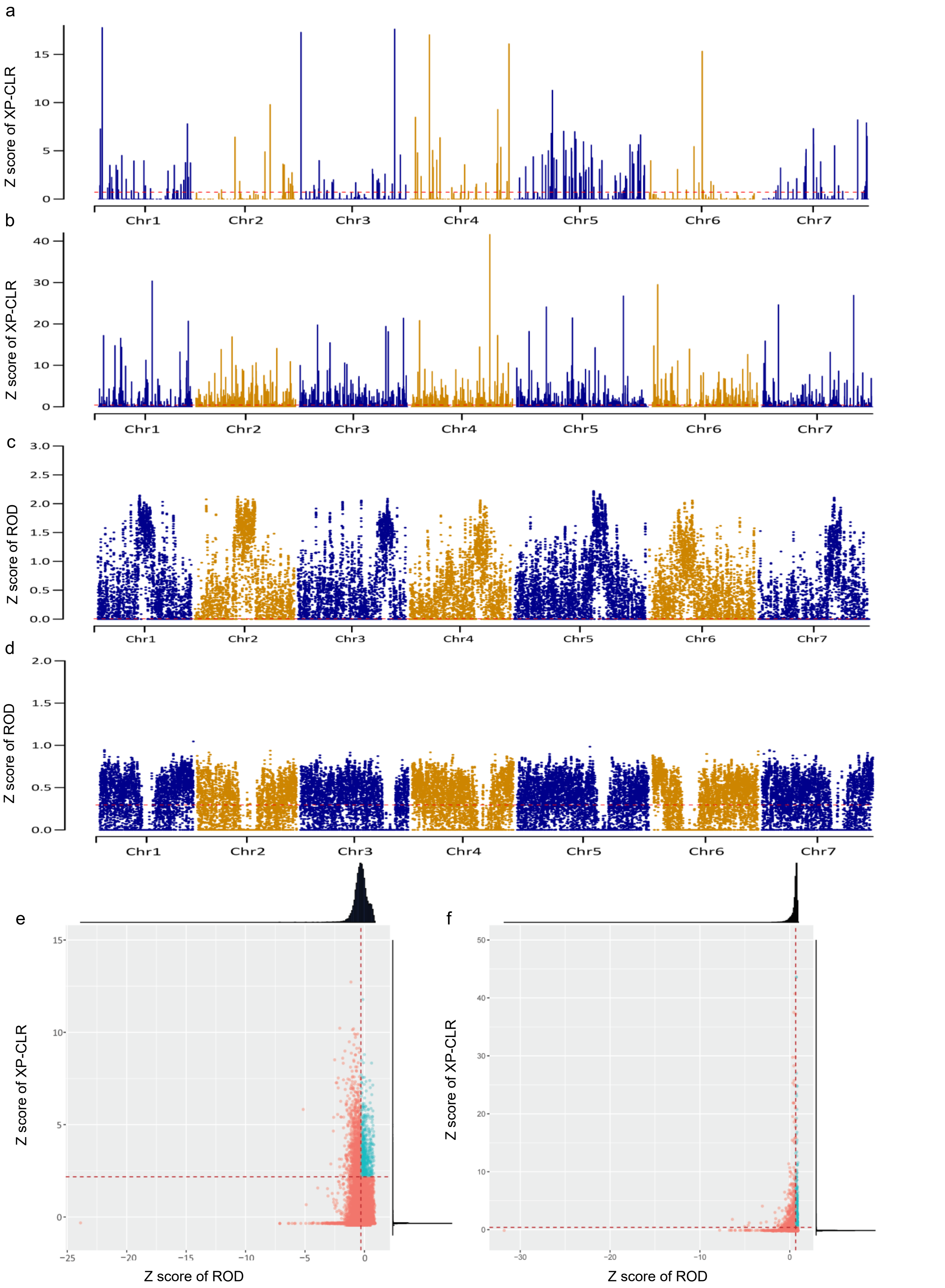

Supplementary Figure 12 Genome wide screening for selective regions (a, b, c, and d) and candidate genes (e and f) using XP-CLR and ROD analyses during pea domestication. a, c, and e = *P. fulvum* vs. *P. sativum*; b, d, and f = *P. fulvum* vs. *P. abyssinicum*. The red dotted lines indicate the top 5% highest XP-CLR values and the top 50% of ROD values.

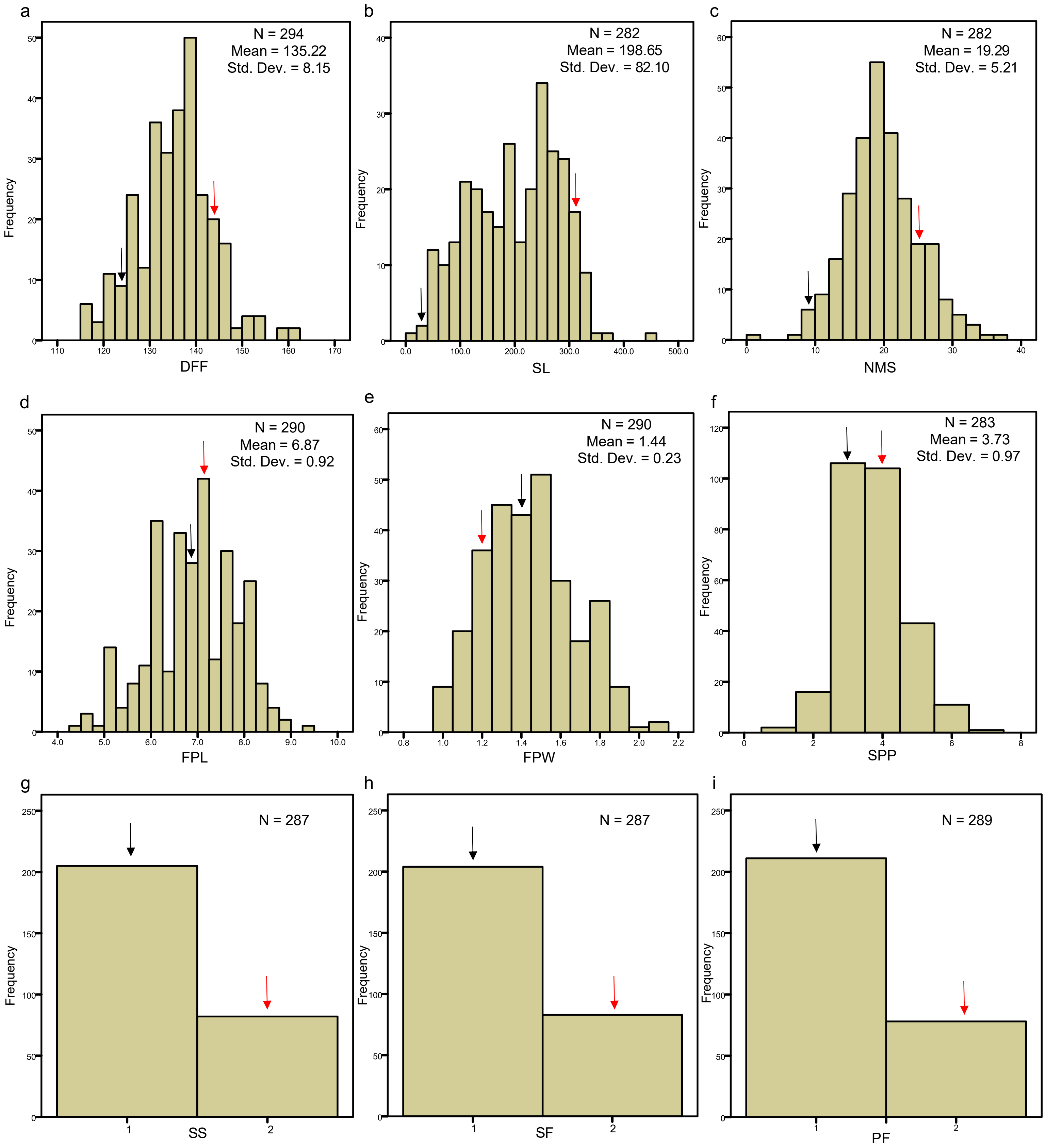

a
N = 294
Mean = 135.22
Std. Dev. = 8.15
Frequency
DFF
b
N = 282
Mean = 198.65
Std. Dev. = 82.10
Frequency
SL
c
N = 282
Mean = 19.29
Std. Dev. = 5.21
Frequency
NMS
d
N = 290
Mean = 6.87
Std. Dev. = 0.92
Frequency
FPL
e
N = 290
Mean = 1.44
Std. Dev. = 0.23
Frequency
FPW
f
N = 283
Mean = 3.73
Std. Dev. = 0.97
Frequency
SPP
g
N = 287
Frequency
SS
h
N = 287
Frequency
SF
i
N = 289
Frequency
PF

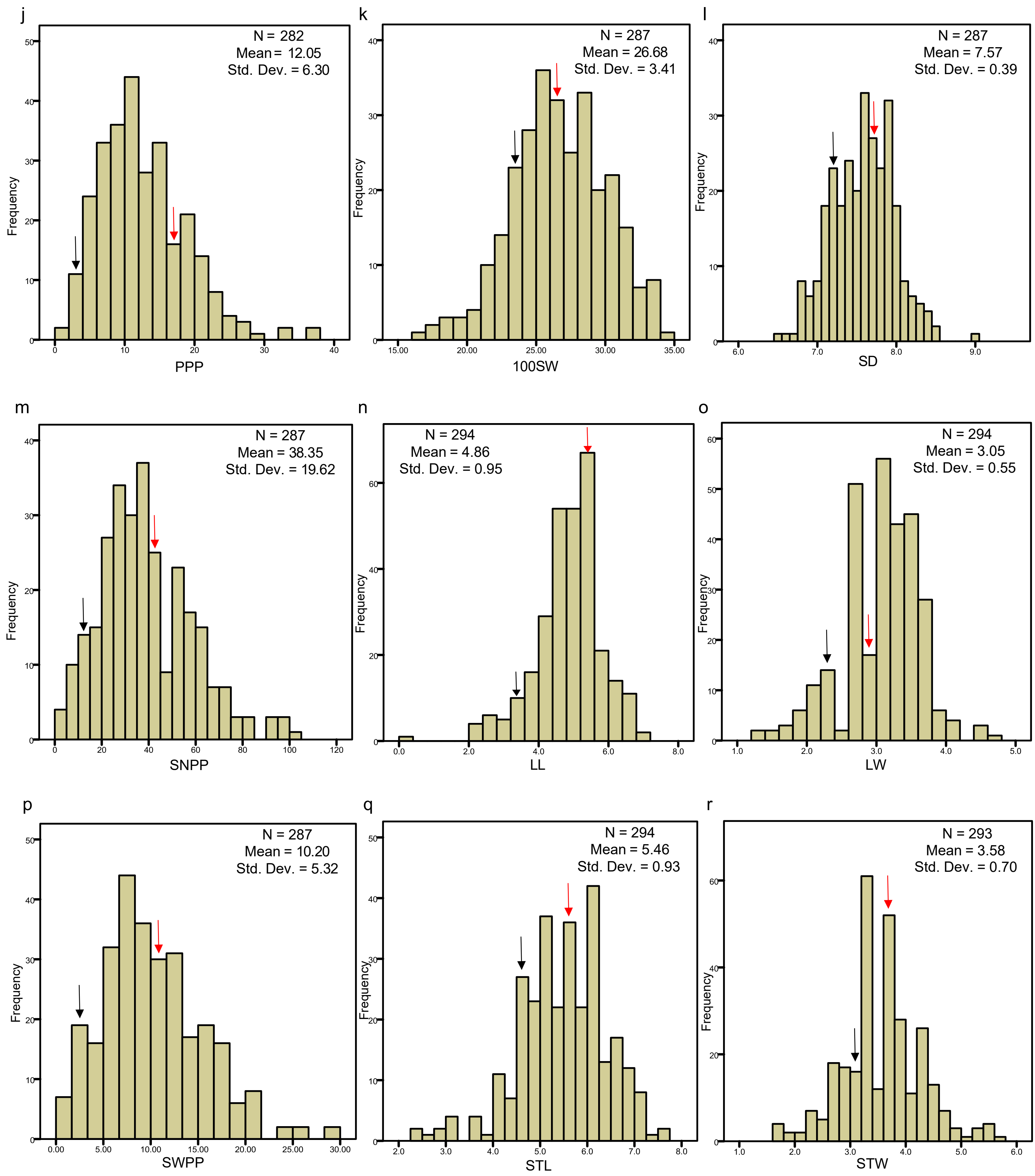

Supplementary Figure 13 Frequency distribution of 18 agronomic traits in 298 F_2 population of pea (a-r). Black and red arrows indicate the parents (WJ and ZW6) of the F_2 population, respectively. (a-r): DFF = days to first flowering, SL = stem length, NMS = nodes of main stem, FPL = fresh pod length, FPW = fresh pod width, SPP = seeds per pod, SS = seed shape, SF = seed form, PF = pod form, PPP = pods per plant, 100SW = 100-seed weight, SD = seed diameter, SNPP = seed number per plant, LL = leaflet length, LW = leaflet width, SWPP = seed weight per plant, STL = stipule length and STW = stipule width.

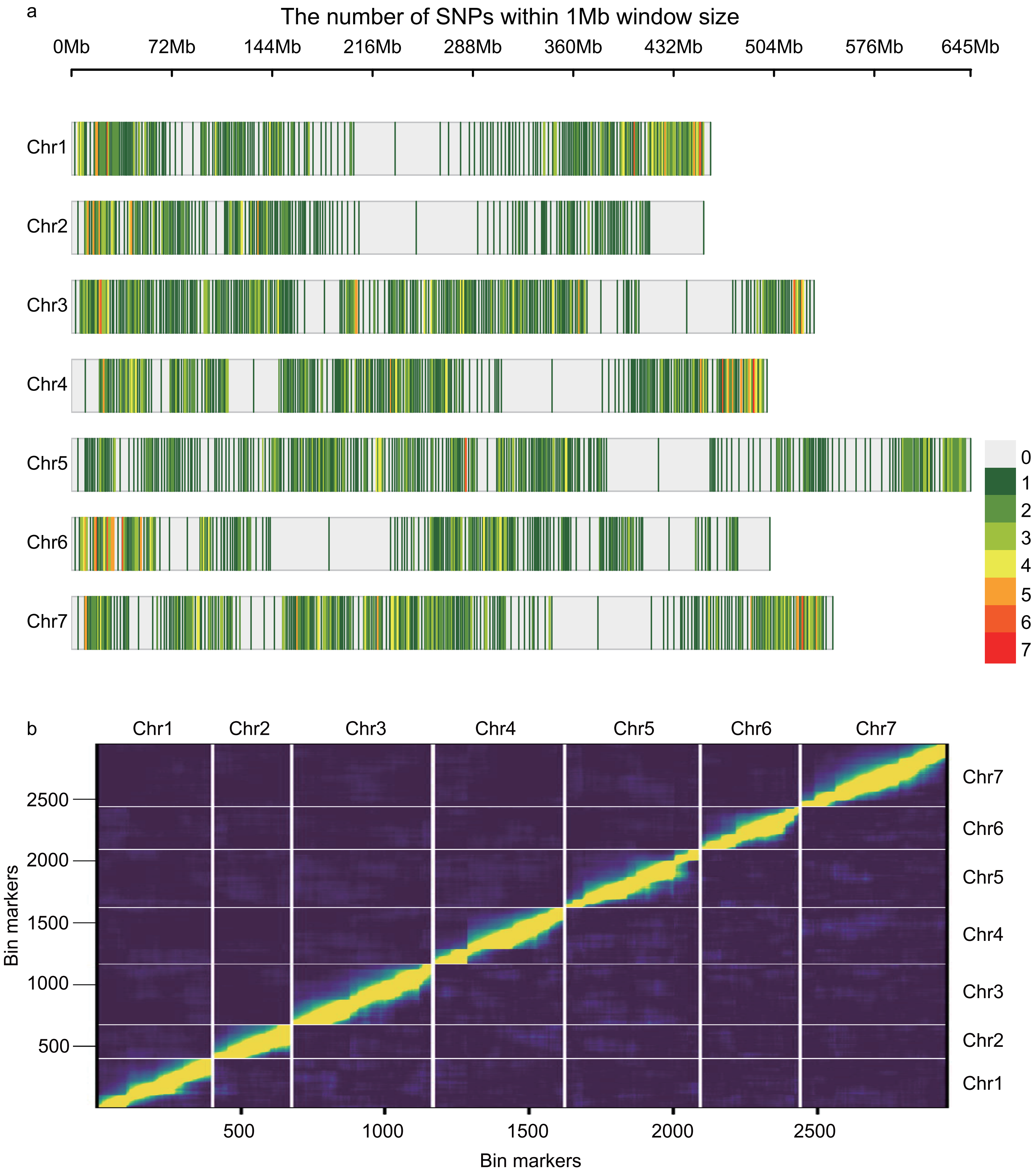

Supplementary Figure 14 Genome distribution (a) and estimated recombination fractions (b) for 2950 bin markers.

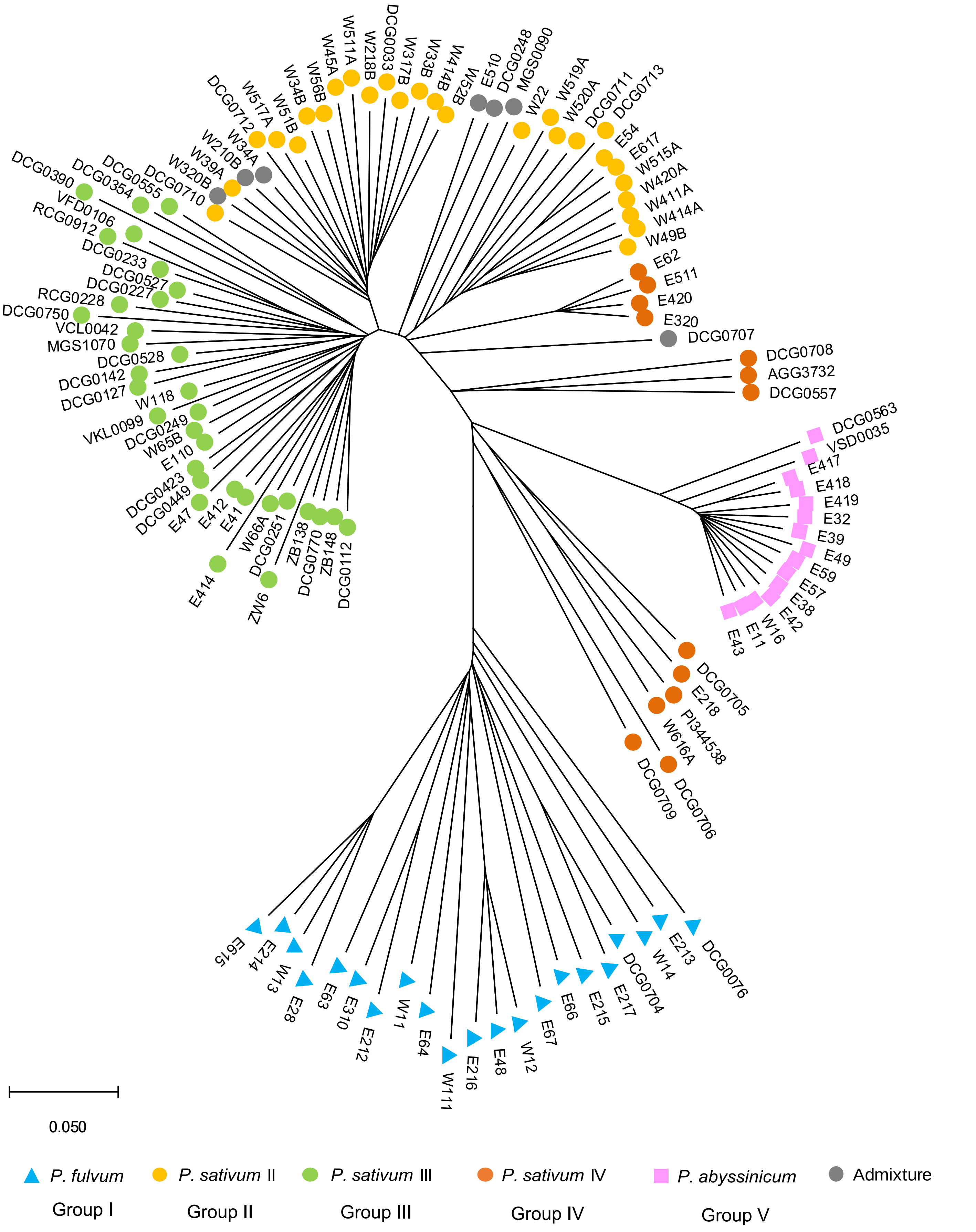

Supplementary Figure 15 Neighbor-joining phylogenetic tree of 116 *Pisum* accessions (including ZW6) based on 112,776 PAVs identified in pan-genome.

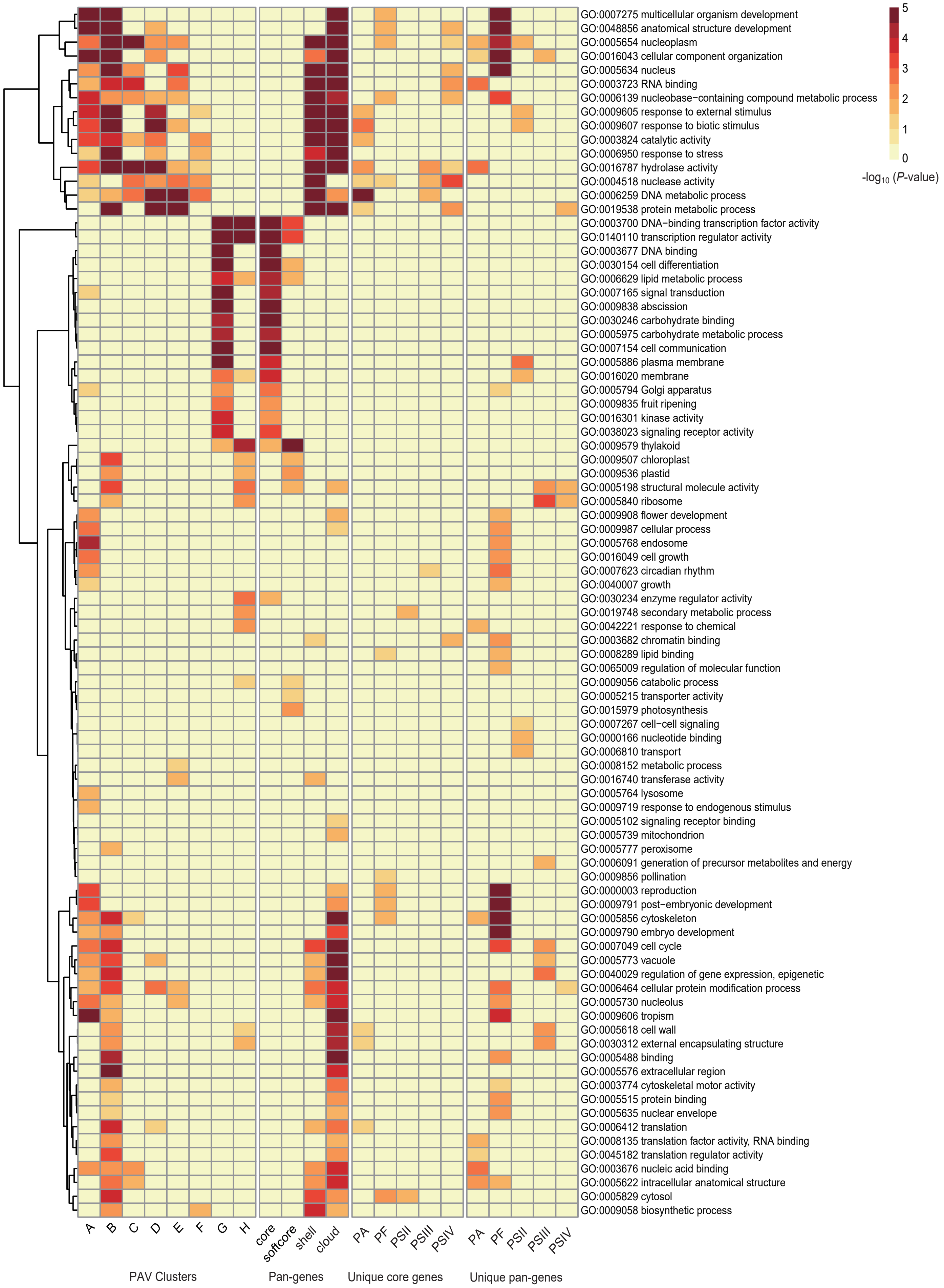

Supplementary Figure 16 GO enrichment analyses of eight PAV clusters as well as pan-genes, unique core genes and unique pan-genes identified in pan-genome of 116 Pisum accessions. PA = *P. abyssinicum*, PF = *P. fulvum*, and PS = *P. sativum*. One-sided Fisher's exact test was applied and p-values were adjusted using Benjamini-Hochberg (BH) method.

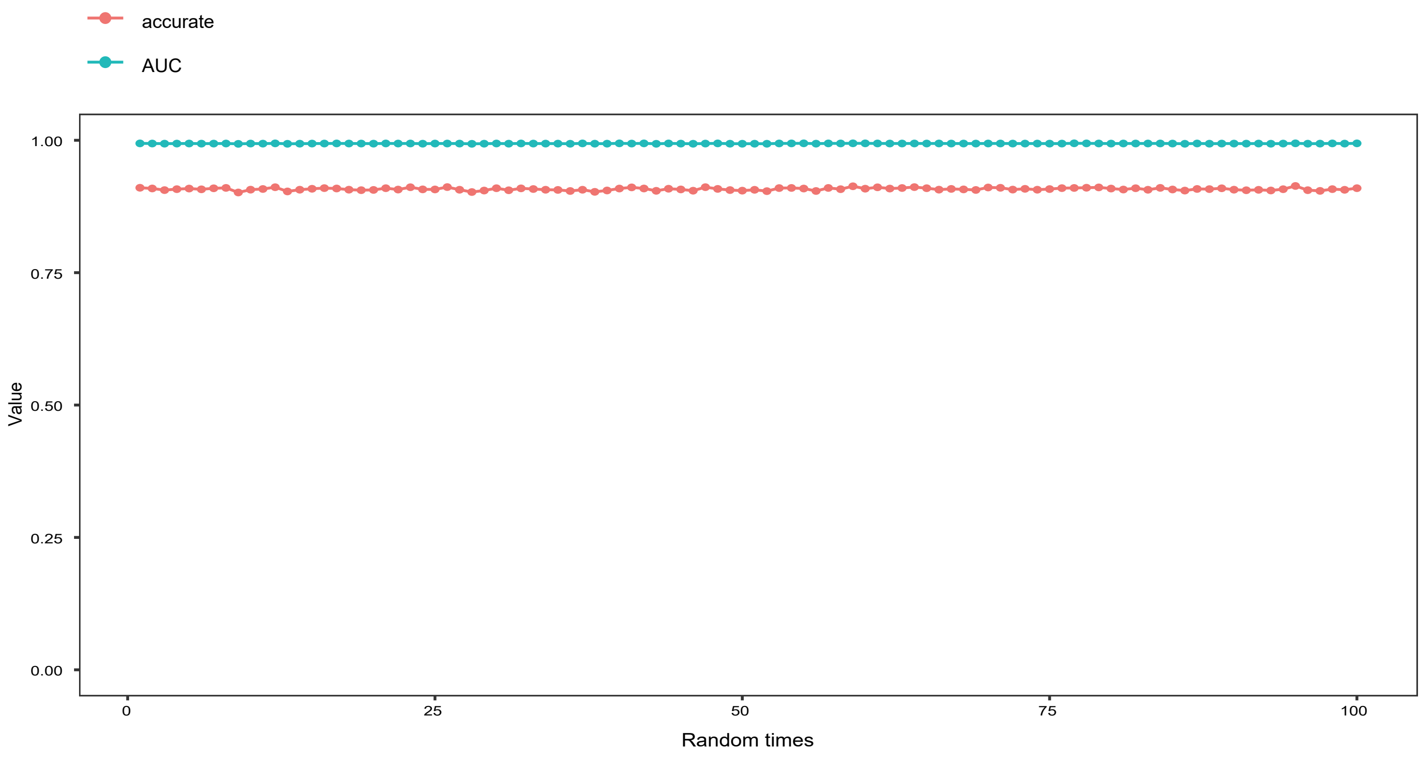

Supplementary Figure 17 Permutation of randomForest to re-assign the 112,776 HOGs into pre-built 8 groups. Red and blue indicate the average accurate and AUC values in 100 runs, respectively.

Supplementary Tables

Supplementary Table 1 Genome size estimation of PeaZW6 with tomato as standard sample by flow cytometry

Species	Peak value of R1	Peak value of R2	Peak value of R3	Mean value ± SD	Genome size (Mb)
**Tomato**	77.10	76.37	77.18	76.88±0.446	800.00
**PeaZW6**	412.84	408.13	413.25	411.41±2.845	4281.06

Supplementary Table 2 Summary of data used for genome assembly and annotation

Sequencing platform	Insert length	Sequencing model	Reads number	Total length
PacBio Sequel	>20kb	SMRT	29.24M	379.34 Gb
Hiseq X	300bp	2x150	6.86G	1031.25 Gb
10X Genomics + Hiseq 2500	NA	2x150	1.63G	244.65 Gb
BioNano Saphyr	NA	NA	1.55M	453.2 Gb
HiSeq	NA	2x150	2.93G	439.3 Gb
RNA-Seq on Hiseq 2000	>300nt	2x100	317.85M	32.1 Gb

Supplementary Table 3 The sequence coverage distribution of the genome

Depth	Pacbio (bp)	Pacbio (%)*	Illumina (bp)	Illumina (%)*
> 0	3,785,468,133	99.97	3,773,560,079	99.66
⩾ 10	3,771,772,158	99.61	3,760,750,912	99.32
⩾ 20	3,763,952,137	99.41	3,754,643,426	99.16
⩾ 30	3,756,250,445	99.20	3,749,325,758	99.02
⩾ 50	3,733,192,867	98.59	3,739,566,400	98.76
⩾ 100	1,087,996,765	28.73	3,714,697,054	98.10
⩾ 200	18,087,964	0.48	3,564,844,390	94.15

* The contig length without gap for PeaZW6 is 3,786,461,935 bp.

Supplementary Table 4 Assessment of genome completeness in pea genome using BUSCO

Searching model	Protein categories	BUSCO			
		PeaZW6		PeaCaméor	
		Number	Percentage	Number	Percentage
**Genome**	Complete BUSCOs (C)	1604	99.38	1562	96.78
	Complete and single-copy BUSCOs (S)	1537	95.23	1518	94.05
	Complete and duplicated BUSCOs (D)	67	4.15	44	2.73
	Fragmented BUSCOs (F)	6	0.37	40	2.48
	Missing BUSCOs (M)	4	0.25	12	0.74
	Total BUSCO groups searched	1614	100.00	1614	100.00
**Protein**	Complete BUSCOs (C)	1578	97.77	1517	93.99
	Complete and single-copy BUSCOs (S)	1516	93.93	1481	91.76
	Complete and duplicated BUSCOs (D)	62	3.84	36	2.23
	Fragmented BUSCOs (F)	15	0.93	67	4.15
	Missing BUSCOs (M)	21	1.30	30	1.86
	Total BUSCO groups searched	1614	100.00	1614	100.00

Supplementary Table 5 The summary of RNA-seq datasets used in this study

Library	Tissue	Raw Reads	Raw Base	High quality rate[1]	Mapping rate[2]
WD-7-23	Root	33,940,496	3,427,990,096	93.04	97.97
WD-8-25	Stem	37,602,558	3,797,858,358	94.76	99.57
WD-B-27	Leaf	41,401,626	4,181,564,226	96.40	99.53
WD-P-11	Immature seed	43,207,910	4,363,998,910	99.59	99.73
WD-PF-21	Flower	43,110,052	4,354,115,252	97.85	99.50
WD-PFU-22	Flower bud	39,698,838	4,009,582,638	97.67	99.59
WD-PP-20	Green pod	36,201,218	3,656,323,018	96.71	99.77
WD-PT-1	Tendril	42,692,044	4,311,896,444	97.40	99.49

[1]High quality rate is the percentage of > 30nt clean reads after trimming adapter sequence and low-quality terminal base

[2]Mapping rate is the percentage of mapped high quality reads with > 50 nt aligned length and > 90% identity using Blat

Supplementary Table 6 Merqury analysis of PeaZW6 and PeaCaméor

Assembly	k-mers uniquely found only in the assembly	k-mers found in both assembly and the read set	QV	Error Rate (%)	solid k-mers in the assembly	Total solid k-mers in the read set	Completeness (%)
**PeaZW6**	2,832,546	3,786,410,593	44.48	0.0036	999,035,347	1,025,354,019	97.43
**PeaCaméor**	234,931,999	3,153,805,448	24.34	0.3680	971,124,901	1,023,752,156	94.86

Supplementary Table 7 A comparison of genome structure between the Medicago and pea genomes

Catalogs		Medicago/PeaZW6	Medicago/PeaCaméor
Reciprocal best hit gene pairs		18,305	18,941
Number of collinear blocks	s10-m30	125	151
	s30-m30	79	48
	s30-m50	62	42
	s50-m50	48	26
Gene pairs in collinear blocks	s10-m30	15,275	8,869
	s30-m30	14,506	6,956
	s30-m50	14,801	8,111
	s50-m50	14,188	7,333

"s" is the abbreviation for MATCH_SIZE, and means the number of genes required to call a collinear block.

"m" is the abbreviation for MAX_GAPS, and means the maximum gaps allowed.

Supplementary Table 8 Assessment of LTR-RT completeness of pea genome using LAI.

	PeaZW6				PeaCaméor			
**Chr**	**Length（bp）**	**Intact LTR-RTs content (%)**	**Total LTR-RTs content (%)**	**LAI**	**Length（bp）**	**Intact LTR-RTs content (%)**	**Total LTR-RTs content (%)**	**LAI**
Chr1	463,644,500	10.79	77.56	13.91	372,165,146	1.30	57.49	2.26
Chr2	492,729,000	10.31	81.69	12.62	427,597,918	1.25	59.62	2.09
Chr3	535,674,373	10.45	80.86	12.92	437,560,801	1.18	58.13	2.03
Chr4	501,613,000	10.71	79.11	13.54	446,350,677	1.16	58.20	1.99
Chr5	652,929,960	10.76	79.70	13.50	579,269,071	1.30	59.13	2.19
Chr6	523,395,090	10.46	78.81	13.28	480,417,049	1.33	57.56	2.31
Chr7	549,106,000	10.59	79.03	13.40	491,380,962	0.98	55.87	1.76
**Total**	**3,719,091,923**	**10.58**	**79.54**	**13.31**	**3,234,741,624**	**1.21**	**58.00**	**2.09**

Supplementary Table 9 Summary of pea protein-coding genes

Features	PeaZW6	PeaCaméor
Gene number	47,526	44,756
Gene total length (Mb)	121.08	124.59
Gene average length (bp)	2,563.70	2,284.10
Gene density within assembly (per Mb)	12.51	11.41
All transcripts	67,527	57,835
CDS average length (bp)	1,122.30	1,015.61
Average exon length (bp)	224.66	308.5
Exons per transcript	4.95	4.33

Supplementary Table 10 Annotation statistics of 47,526 predicted proteins encoded in the PeaZW6 genome

Evidence	Proteins	Percentage(%)
eggNOG	43,076	90.64
Pfam	27,318	57.48
KEGG	20,110	42.31
Gene Ontology(GO)	20,322	42.76
NCBI nr	43,578	91.69
UniProtKB/Swiss-Prot	26,537	55.84
**With any annotation evidences**	**45,362**	**95.45**

Supplementary Table 11 Types and regions of SNPs and small InDels identified in the 118 cultivated and wild pea accessions

Type	SNP		InDel	
	Count	Percent	Count	Percent
Downstream	5,343,305	14.1%	448,280	18.8%
Exon	909,202	2.4%	25,639	1.1%
Intergenic	24,293,585	64.1%	1,261,165	53.0%
Intron	2,012,387	5.3%	255,404	10.7%
Splice_site_acceptor	1,532	0.0%	896	0.0%
Splice_site_donor	1,354	0.0%	649	0.0%
Splice_site_region	55,335	0.1%	7,019	0.3%
Upstream	5,310,378	14.0%	379,340	15.9%

Supplementary Table 12 Size and types of SVs identified in the 118 cultivated and wild pea accessions

SV type	Items	30-50	50-500	500-5000	>5k	Total
**DEL**	Count	157,967	131,960	34,772	30,864	355,563
	Percentage	95.5%	95.7%	91.1%	88.6%	94.5%
**DUP**	Count	1	5,412	3,392	3,984	12,789
	Percentage	0.0%	3.9%	8.9%	11.4%	3.4%
**INS**	Count	7,402	555	0	0	7,957
	Percentage	4.5%	0.4%	0.0%	0.0%	2.1%
**TOTAL**	Count	165,370	137,927	38,164	34,848	376,309
	Percentage	43.9%	36.7%	10.1%	9.3%	100.0%

Supplementary Table 13 GO terms enriched in unique and common genes in domestication sweeps from *P. fulvum* to *P. sativum* compared to those from *P. fulvum* to *P. abyssinicum*

GO term ID	GO term name	Category	*P* value[a]	Adjusted *P* value[a,d]	*P* value[b]	Adjusted *P* value[b,d]	*P* value[c]	Adjusted *P* value[c,d]
GO:0004518	nuclease activity	Molecular function	0.0072	0.1882				
GO:0005102	signaling receptor binding	Molecular function	0.0118	0.1537				
GO:0000166	nucleotide binding	Molecular function	0.0133	0.1150				
GO:0005488	binding	Molecular function	0.0173	0.1126				
GO:0003677	DNA binding	Molecular function			0.0313	0.7821		
GO:0008135	translation factor activity, RNA binding	Molecular function			0.0401	0.5018		
GO:0045182	translation regulator activity	Molecular function			0.0416	0.3470		
GO:0008219	cell death	Biological process			0.0328	1.0000		
GO:0006629	lipid metabolic process	Biological process			0.0343	0.6175		
GO:0009628	response to abiotic stimulus	Biological process					0.0001	0.0021
GO:0009416	response to light stimulus	Biological process					0.0021	0.0410
GO:0009607	response to biotic stimulus	Biological process					0.0030	0.0399
GO:0007165	signal transduction	Biological process					0.0039	0.0388
GO:0007154	cell communication	Biological process					0.0100	0.0798
GO:0009605	response to external stimulus	Biological process					0.0211	0.1410

a, *P* value of GO enrichment for the 5,785 candidate selected genes unique to *P. sativum*.

b, *P* value of GO enrichment for the 1,494 candidate selected genes common to *P. sativum* and *P. abyssinicum*.

c, *P* value of GO enrichment for the 8,638 candidate selected genes unique to *P. abyssinicum*.

d, One-sided Fisher's exact test was applied and *P* value were adjusted using Benjamini-Hochberg (BH) method.

Supplementary Table 14 Summary of the genetic linkage map in pea

Chr	Number of bin markers	Map length (cM)	Average of marker spacing	Maximum of marker spacing
1	400	123.1	0.31	3.60
2	275	71.0	0.26	2.72
3	491	144.7	0.30	8.30
4	456	139.1	0.31	20.22
5	471	168.7	0.36	6.25
6	348	129.7	0.37	15.39
7	509	147.8	0.29	6.81
**Total**	**2950**	**924.1**	**0.31**	**20.22**

Supplementary Notes

Table of Contents

1. Sequencing, assembly and evaluation of PeaZW6
1.1 Hi-C experiment
1.2 Genome assembly of PeaZW6
1.3 Quality assessment of PeaZW6
2. Repeat and gene annotation of PeaZW6
3. Genomic polymorphism and population genetics
4. QTL analysis and candidates of Mendel's genetic loci
5. *Pisum* pan-genome assembly, annotation and PAV analysis
References

1. Sequencing, assembly and evaluation of PeaZW6

1.1 Hi-C experiment

Fresh leaves were fixed with 1% formaldehyde solution in MS buffer composed of 10 mM potassium phosphate, pH 7.0; 50 mM NaCl; 0.1M sucrose for 30 min and incubated in MC buffer with 0.15 M glycine for 5 min at room temperature under vacuum, respectively. Fixed tissue was homogenized with liquid nitrogen for nuclei isolation and filtered with a 40-nm corning® cell strainer (Corning Incorporated, USA). Chromatin was digested for 16 h with *Hind*III restriction enzyme (New England Biolabs, USA) at 37 °C. DNA ends labeled with biotin were incubated at 37 °C for 45 min, and then treated with 20% SDS solution to inactivate the enzyme. DNA ligation was performed using T4 DNA ligase (New England Biolabs, USA) and incubation at 16°C for 4-6 h. After ligation, proteinase K (Promega, USA) was added to reverse cross-linking during incubation at 65 °C overnight. DNA fragments were purified and dissolved in 86μL de-ionised water. Purified DNA was fragmented to a size of 300–500 bp, and DNA ends were then repaired. DNA fragments labeled by biotin was finally separated on Dynabeads® M-280 Streptavidin (Life Technologies, USA).

1.2 Genome assembly of PeaZW6

A total of 379.34 Gb long reads generated by PacBio Sequel platform (Pacific Biosciences, Menlo Park, CA, USA) were *de novo* assembled using CANU (v1.8)[1]. The parameters were optimized for highly repetitive genomes according to CANU documentation (mhapSensitivity=normal, minOverlapLength=1000, correctedErrorRate=0.050, batOptions=-dg 3 -db 3 -dr 1 -ca 500 -cp 50). The initial CANU assembly was corrected using a combination of PacBio long and Illumina short reads with Pilon (v1.23)[2] using default parameters. Potential duplicated or haploid contigs were purged using PurgeHaplotigs (v1.1.1)[3]. The purged contigs were further scaffolded with 10X Genomics data using ARCS (v1.0.4)[4] and LINKS (v1.8.6)[5]. The 10X scaffolds were then mapped with a DLE1 labelled BioNano optical map using BioNano Solve package (v3.4_06042019a) to be elevated to super-scaffolds. Local assembly errors were simultaneously corrected by BioNano Solve. The super-scaffolds were then mapped with a Hi-C library (HindIII digested) and anchored into

chromosome level scaffolds using Juicer (v1.5.6)[6] and 3d-dna pipeline (v180922)[7]. The assembly files generated were visualized and manually corrected and optimized using built-in assembly tool JuiceBox Assembly Tools (JBAT) of Juicebox (v1.11.08)[8], breaking weak or ambiguous contact links between large TAD blocks and rebuilding the boundaries. The Hi-C scaffolding-based assembly was evaluated and anchored to chromosomes using ALLMAPS (v1.0)[9] with genetic markers from a previous study[10].

For the chloroplast genome, we used NC_014057.1 from RefSeq as reference and searched the assembled contigs using BLAST(v2.5.0+)[11] to find the circular contig 'HiC_scaffold_24' as the candidate, which was manually finished and renamed to 'chrP'. For the mitochondrion genome, since no complete reference was available, we used available mitochondrion genes from NCBI as seed to search the contigs for mitochondrion candidates using BLAT[12], to find the circular contig 'tig00000765' as the candidate, which was also manually finished and renamed to 'chrM'. Other basic sequence manipulation and statistics were completed using SeqKit (v0.15.0)[13]. The assembly PeaZW6 is available at Pea Genome Database (https://www.peagdb.com/) containing the following module: "Home", "Jbrowse", "Blast", "Pathway", "Search", and "Download".

Pea is one of the most important legume crops in the agricultural ecosystems, and has contributed immensely to advances in plant genetics[14]. However, contig assembly in the previously reported draft reference genome for pea was mainly based on NGS, with low-coverage PacBio data used for gap-filling, which resulted in fragmented assemblies, especially in complex repeat regions. The N50 values of the scaffolds and contigs were only 415.9 Kb and 37.9 Kb, respectively[15,16], an inadequate assembly quality for state-of-the-art studies. In this study, using a combination of SMRT sequencing, BioNano optical mapping and Hi-C scaffolding, the novel assembly showed obvious improvements in the consistency of the genetic map in comparison to the previous reference genome. After mapping the corrected PacBio reads and NGS reads to the PeaZW6 assembly, it was found that 99.41% and 99.16% of the assembly was covered by at least 20 PacBio reads and 20 NGS reads, respectively, which confirmed the high quality of PeaZW6 and indicated the consistency of mapping rate of the NGS sequences and that of the PacBio sequencing strategy, even when sequencing a large and complex plant genome (Supplementary Table 3, Supplementary Figure 6). To further illustrate the structural accuracy of the genome structure of the PeaZW6 assembly, syntenic regions were detected using orthologous gene pairs between the pea genome and Medicago (*Medicago truncatula*), the most closely related species with a complete genome sequence assembly available. It was obvious that the number of homologous genes within the syntenic regions of PeaZW6/Medicago was obviously and consistently greater than that in PeaCaméor/Medicago with different parameters (Supplementary Table 7), validating the long continuousness of the PeaZW6 assembly.

1.3 Quality assessment of PeaZW6

The gene completeness of the ZW6 and previous Caméor v1a assembly[15] were assessed with Benchmarking Universal Single-copy Orthologs (BUSCO) (v5.0.0)[17], in

genome mode using 'embryophyta_odb10' model. The K-mer completeness and heterozygosity of PeaZW6 and PeaCaméor were evaluated by Merqury[18], which was analyzing the K-mer spectrums (K=21) generated from the assemblies and their corresponding raw NGS sequencing data. For the mapping summary and statistics, the raw NGS reads were mapped using BWA-MEM (v0.7.15)[19] and the corrected PacBio reads were mapped using Minimap2 (v2.1)[20] using default parameters, respectively. The quality of repetitive genomic regions was assessed using the LTR Assembly Index (LAI)[21] as follows: (1) LTRharvest[22] (with parameters --similar 85.00 --vic 10 --seed 30 --seqids yes --motif TGCA --motifmis 1 --minlenltr 100 --maxlenltr 3,500 --mindistltr 1,000 --maxdistltr 20,000 --mintsd 4 --maxtsd 20) and LTR_FINDER[23] (with parameters: --l 100--L 3,500 --d 1,000 --D 20,000 --M 0.3) were used to *de novo* predict the candidate LTR-RTs (full-length LTRs retrotransposon) in the two pea assembly sequences. (2) LTR_retriever[24] was then used to combine and refactor all the candidates to get the final full-length LTR-RTs. LAI was calculated based on the formula: LAI= (Intact LTR-RTs length/total LTR-RTs length) × 100.

2. Repeat and gene annotation of PeaZW6

Repetitive sequences and families were identified using RepeatModeler and RepeatMasker (http://repeatmasker.org/). RepeatModeler was employed to build a ZW6-specific library of classified repeat family by sampling and clustering repeat sequences from the PeaZW6 assembly. The library was used by RepeatMasker to identify and mask repetitive sequences into lower case in the PeaZW6 and PeaCaméor assemblies, with default parameters. The full-length LTR_retrotransposon was identified by LTR_FINDER_parallel (v1.0.7) with default parameters[23,25].

Protein-coding genes were annotated using a combination of *ab initio* gene prediction, homology-based gene prediction and transcriptome-based prediction. A total of 71 RNA-seq libraries were used to construct transcripts, including 8 libraries from ZW6 in this study and 63 libraries from public databases. The RNA-seq data were mapped using HISAT2 (v2.1.0)[26] and transcripts were constructed using StringTie (v1.3.4)[27]. All constructed transcripts were combined using TACO (v0.7.3)[28] for a high quality non-redundant set. The ORFs on the transcripts were extracted with TransDecoder in the PASA pipeline (v5.5.0)[29]. The complete ORFs from TransDecoder were used as the training set for *ab initio* prediction performed by BRAKER2 pipeline (v2.1.5)[30]. For homology-based prediction, protein sequences collected from closely related species and published legume genomes were mapped to the PeaZW6 genome using GenomeThreader (v1.7.1)[31]. The automated annotation pipeline and toolkit Funannotate (v1.7.4) (https://funannotate.readthedocs.io/en/latest/index.html)[32] was used to combine the results from different evidences for a preliminary annotation set containing 123,623 gene loci. Since the number was almost tripled to common plant genomes, we further applied a multi-level curation workflow to reduce potential false predictions. The preliminary results from Funannotate was the Level 1. In Level 2, protein domains were identified by HMMER (v3.3.1)[33] against PFAM database (v31)[34], and genes with retrotransposon functional domain were removed, retaining 102,782 loci. In Level 3, single exon genes with only *ab initio* prediction evidence without

homologous or expressions were removed (possibly introduced by retrotransposons), retaining 69,576 loci. In Level 4, homology-based search was performed by BLASTP (v2.5.0+) (E-value < $1e^{-3}$)[11] towards UniProtKB/SwissProt[35], NR and KEGG[36] databases, as well as protein sequences used in homology-based prediction. Genes without any homology to these databases were removed. Finally, frameshifted and partial genes were removed using the GFFRead (v0.11.6) tool from Cufflinks[37]. Finally, a total of 47,526 gene loci were retained for further analysis. Functional annotation was performed using InterProScan (v5.0)[38] and eggNOG-mapper (v2.1.6)[39] to identify their potential functions. In addition, BLASTP (v2.5.0+) (E-value < $1e^{-3}$) was also used to search public databases, including NR and KEGG, for annotation rate and other cross checking. Note that, the gene length used in statistics was defined as the chromosomal distance between the start codon and stop codon. For chloroplast and mitochondrion, the *ab initio* prediction and ORF extraction was done using genetic code 11.

By using a combination of *ab initio* predictions, homology-based mapping and deep transcriptome sequencing from multiple tissues of *P. sativum*, a total of 47,526 coding genes were identified in PeaZW6 (Supplementary Tables 9 and 10). The average exon length was 224.66 bp. The mean number of exons per gene was 4.95 per gene (Supplementary Table 9).

3. Genomic polymorphism and population genetics

To investigate genomic polymorphisms in cultivated and wild pea within *Pisum*, five seeds of 76 accessions representing different taxa of *Pisum* including 21 of *P. fulvum*, 13 of *P. abyssinicum* and 42 of *P. sativum*[40] were planted in glasshouse under natural conditions of the Institute of Crop Sciences (ICS), Chinese Academy of Agricultural Sciences (CAAS), Beijing in 2020. SNPs, InDels and SVs were mined using new resequencing data from the 76 representative genotypes of cultivated and wild pea with an average sequence coverage of 14.98 X and public data including 42 *Pisum* accessions with an average sequence coverage of 11.77 X[15] (Supplementary data 2).

SNPs and InDels were first filtered with the GATK recommended variant filtration, and then filtered using VCFtools (v0.1.15)[41] with the following parameters: --max-missing 0.8 --minQ 30 --minDP 5 --maf 0.05 --min-alleles 2 --max-alleles 2 to obtain the final high quality SNPs and InDels for further population genetic analyses. We detected SVs separately for each pea strain and explored these SVs from the population perspective. The SVs called from >= 2 accessions were used for further analysis. This type of population-filter would greatly improve the accuracy and sensitivity of short-reads-based SVs calling[42]. The combined SVs from all pea cultivars were further filtered using VCFtools (v0.1.15)[41] with the following parameters: --max-missing 0.5 --maf 0.01 to make sure the accuracy of SVs.

For the small InDels, more deletions (63.6%) than insertions (36.4%) were detected. In addition, a curated set of 376,309 SVs larger than 30 bp was called from 118 *Pisum* accessions, and comprised 355,563 deletions (DEL, 94.5%), 12,789 duplications (DUP, 3.4%), and 7,957 insertions (INS, 2.1%). The length distribution of the SVs was as follows: 30-50 bp, 165,370, 43.9%; 50-500 bp, 137,927, 36.7%; 500-5000 bp, 38,164, 10.1%; >5k, 34,848, 9.3% (Supplementary Table 12), which indicated

that most SVs were small.

To clarify the phylogenetic relationship and population genetic structure of cultivated and wild peas within *Pisum*, ADMIXTURE was applied to both SNP and SV datasets with the number of groups (K) ranging from 2 to 10, and the results produced for the two datasets were highly consistent (Fig. 4b, c, Supplementary Figure 9). Different types of variants revealed a consistent pattern of three species in *Pisum* and calls into question the hypothesis that cultivated *P. sativum* subsp. *sativum* and *P. abyssinicum* were independently domesticated from the distinct *P. s. elatius*[15]. These results resolved a long-standing controversy about the species classification of *Pisum*, providing new insights to pea domestication[15,40,43,44].

Two homologous genes *Psat02G0081200* and *Psat02G0507900* corresponding to *GmHs1-1*[45]and *GmG*[46], respectively, were identified in PeaZW6, and both were present in the putative selected region of *P. abyssinicum.* Interestingly, a significant reduction in the nucleotide polymorphism was observed for *Psat02G0081200* from *P. fulvum* to *P. abyssinicum*, indicating positive selection, while more polymorphisms were preserved for *Psat02G0507900* in *P. abyssinicum* than in *P. fulvum* implying balancing selection (Supplementary data 5). Another candidate gene of seed dormancy for pea was a homologue of the *KCS12* gene from *M. truncatula*, which controls seed coat formation[47].

4. QTL analysis and candidates of Mendel's genetic loci

To explore the genetic basis of important agronomic traits in the pea, 18 agronomic traits were phenotyped in a population (300 F_2) derived from a cross between the WJ (tall plant, wrinkled seeds and constricted pods) and ZW6 (dwarf plant, pitted seeds and inflated pods) strains (Supplementary data 6; Supplementary Figure 13). The entire population was genotyped using GBS. Raw SNPs were first filtered with the GATK recommended variant filtration, and then filtered using VCFtools (v0.1.15)[41] with the following parameters: --max-missing 0.8 --maf 0.05 --min-alleles 2 --max-alleles 2 to obtain the final high quality SNPs required to construct the genetic linkage map. The final VCF file was converted into ABH-format mapping data file using the Perl script run_pipline.pl in Tassel (v 5.2.40)[48] and screened for suitable markers to construct the genetic linkage map. Non-homozygous parents' variants, markers or individuals with more than 20% missing data, duplicated and significant distorted segregation markers were removed using R/qtl[49]. SNPbinner[50] was used to calculate breakpoints and construct genotype bins with the following parameters: --min-ratio 0.01 in "crosspoints" command and --min-bin-size 5000 in "bins" command. Subsequently, QTL analysis was performed for 12 agronomic traits; six traits that were highly correlated with other traits were excluded (Supplementary data 7).

QTL analysis enabled the rediscovery of two of Mendel's genes and candidate genes for pod form in three major QTLs (Fig. 5, Supplementary data 8-11, Supplementary notes). Combining model plant candidate gene function research findings with homology alignment against the PeaZW6 reference genome[51,52], possible candidate genes for *GP*/*gp*, *V*/*v*, and *Fa*/*fa* were respectively proposed to be homologues of *LCD1* in *Arabidopsis thaliana*, which is involved in differential

development of bundle sheath and mesophyll cell chloroplasts[53-54]; beta-glucosidase12 gene in *Oryza sativa*, which is involved in lignification and cell wall remodeling processes[55]; and *CLV1* in *A. thaliana*, which is involved in controlling shoot and floral meristem size[56-57] (Supplementary data12).

5. *Pisum* pan-genome assembly, annotation and PAV analysis

Each *Pisum* accession was *de novo* assembled from the NGS resequencing data using DBG-based MEGAHIT (v1.2.9)[58] and OLC-based MaSuRCA (v3.4.0)[59] independently. For MEGAHIT, the parameter '--presets meta-sensitive' were set for accessions with ≤10X sequencing depth and '--presets meta-large' for those with > 10X depth. For MaSuRCA, 'USE_LINKING_MATES = 1' was set for better continuity on the sake of longer running time. The assembled contigs from MEGAHIT and MaSuRCA were merged using CD-HIT (v4.8.1)[60] with parameter '-c 0.97 -d 0 -M 0 -g 1 -G 0 -aS 0.50', determined after testing on the tradeoff of completeness, duplication and missing of BUSCO genes. Next, the merged contigs were anchored to the ZW6 reference using RagTag (v2.0.1)[61], using parameter '-f 500 --mm2-params '-x asm10' -w -u', a similar strategy to the Panoramic pipeline[62]. We didn't use Panoramic pipeline directly due to the large size of genome. After a total of 118 assemblies were generated (76 from this study and 42 from public repositories), the qualities of these assemblies were assessed using BUSCO as previously described for PeaZW6 (Supplementary data 13). Note that 3 accessions were excluded from downstream analysis due to their deficiency (C＜90%) in BUSCO completeness after RagTag assembly.

To find out the pan-genes, gene predictions were firstly done on individual genomes. First, repeat sequences were masked to lowercase using RepeatMasker. Then, the BRAKER2 pipeline[30] was used to predict genes on each genome using the pre-trained ZW6 model. The protein sequences from PeaZW6, PeaCaméor and SwissProt database were used as hints to increase the accuracy. Predicted protein sequences were clustered using CD-HIT (v4.8.1)[60] (-c 0.99 -d 0 -M 0 -g 1) to remove duplicated genes. Next, genes overlapping the masked repeat elements above the given cutoff (＞50% length) were removed to avoid potential retrotransposon. Also, genes were aligned to PFAM database using HMMER (v3.3.1)[33] and to UniRef90 database (downloaded on 2021-02-01) using BLASTP (v2.5.0+) (E-value < $1e^{-5}$) to filter out fragmented genes whose length coverage of target sequences was <50%. Finally, the retained genes were used as final annotation for an accession and were aligned to ZW6 genes to determine if they are additional genes using BLASTP (v2.5.0+) (E-value < $1e^{-5}$) (Supplementary Data 14).

By aligning assemblies to the PeaZW6, the length of identified novel sequences to PeaZW6 ranged from 3.38 Mb (ZB138, 0.16%) to 323.52 Mb (E66, 15.36%), and 142.52 Mb in average (7.18%). The number of additional genes to PeaZW6 ranged from 451 (ZB138, 1.10%) to 6,179 (W111, 14.07%), and 2,442 in average (5.93%) (Supplementary data 14). Among genetic groups, the percentage of novel sequences increased as their genetic distance to ZW6 increased; for example, the most distant group (*P. fulvum*) had 15.71% on average, while the closest group (*P. sativum* III) had only 2.74%.

To investigate the presence and absence variations (PAVs) pattern of pan-genes, all proteins from 116 accessions (115 from resequencing plus the ZW6), were clustered using OrthoFinder (v2.5.4)[63] (-I 1.5 for MCL clustering and -y enabled for splitting paralogs) into phylogenetic hierarchical ortho-groups (HOGs) as representative of pea pan-genes. Note that the HOGs and -y switch were introduced to OrthoFinder (v2.5.4 onwards)[63] since v2.5.4 onwards, which test the phylogeny and split the paralog genes into distinct HOGs instead of a classical gene-family oriented ortho-groups (OGs). This feature has enabled it a suitable solution for pan-gene analysis. As the number of genomes increased, the number of core-genes decreased while the number of pan-genes increased, which gradually converged to saturation (Fig. 6a). Finally, genes from 116 qualified genomes (115 from resequencing plus PeaZW6) were clustered into 112,776 phylogenetic hierarchical ortho-groups (HOGs), representatives of pan-genes based on cross-genome phylogeny of orthologues. The PAV patterns of HOGs represented the PAV patterns of non-redundant pan-genes (Fig. 6; Supplementary data 16).

After the HOGs were generated, we further used a 'map-to-pan' strategy to recover falsely missed HOGs in each accession, which could be potentially due to the sequencing bias, the fragmented assembly or the partial gene-predictions. The gene sequences from all accessions were extracted as a reference. The raw genomic data from all 116 accessions were mapped to the reference using Minimap2 (v2.1)[20] with parameter '-Y -N 32768' for alternative hits as many as possible, and the number of mismatches (NM) were limited to NM<=1 using samtools[64] allowing at most 1bp mismatch. Genes covered over 99% length and over 3X depth by sequencing reads were considered present in the accession, and their corresponding HOGs were consequently marked as 'present' in the PAV table.

The final PAV pattern across 116 genomes were determined by integrating the gene count per accession per HOG and the result from map-to-pan strategy (Supplementary data 18). Orphan genes presented in only one genome were removed. The final PAV pattern clustered by 'hclust' package using 'ward.D' method and illustrated by 'pheatmap' package in R (v3.6.0). Based on their percentage of genomes shared by, the HOGs were classified into core genes (⩾99% of genomes), soft-core genes (⩾90% and <99%), shell genes (⩾15% and <90%) and cloud genes (<15%), as definition in Roary[65], for all accessions and genetic groups. In *Pisum* overall, these corresponding numbers were 15,470, 6,170, 41,028 and 50,108, representing 35.19%, 15.54%, 44.28% and 4.99% (Supplementary data 16). Note that the numbers were calculated on clustered genes while the percentage were calculated on total genes before clustering, because the percentage should be representing the situation for a single genome. Within genetic groups, the percentage of core-genes ranged from 43.52% (*P. sativum*, n=64) to 90.79% (*P. sativum* IV, n=4), higher than the *Pisum* overall (Supplementary data 16). Notably, *P. abyssinicum* (75.52%, n=15) showed evident higher core percentage than Admixture (46.72%, n=16) at similar number of accessions, while the *P. sativum* (43.52%, n=64) showed a similar core percentage with *P. fulvum* (44.03%, n=21) at tripled number of accessions. As mentioned in the manuscript, this could suggest that the genetic diversity might also contributed to the percentage of core genes. Meanwhile, the core-genes also tended to be more conserved in 27 other plant genomes (Fig. 6b;

Supplementary data 17), suggesting their roles of fundamental functions. Moreover, the neighbor-joining tree of PAVs also showed clear separation of 116 *Pisum* accessions, which is highly consistent with the results based on SNPs and SVs (Supplementary Figure 15), suggesting the important genetic variations contributed to domestication of *Pisum* were also buried in PAVs.

To inspect the gene preference and functional enrichment of different groups in the pan-genome, HOGs were further clustered by PAV patterns using 'cutree' in R into eight clusters named A to H (Fig. 6c). Notably, the 'hclust' has an upper limit of 65,535 columns for input and exceptionally crashed around 45,000 columns. To overcome the challenge of clustering all the 112,776 HOGs, 40,000 HOGs were first randomly selected to cluster and split into 8 groups as training data. Next, the 'randomForest' package was used to build a classifier and re-assign the 112,776 HOGs into pre-built 8 groups, and the average AUC achieved 0.98 in 100 runs (Supplementary Figure 17). Cluster A comprised mainly genes predominant in *P. fulvum*, while Cluster B comprised mainly cloud-genes in few genomes. Cluster C and E consist mainly of shell-genes absent in *P. abyssinicum*, while genes in E are also preferred by *P. sativum*. Cluster D and F are mainly shell-genes preferred in *P. abyssinicum*, while genes in F are also preferred in *P. sativum*. Cluster G contains mainly core-genes and H contains mainly soft-core-genes. The pattern showed that the *P. fulvum* and *P. abyssinicum* accessions possessed rich unique genes, indicating their potential value as future breeding resources. Many *P. sativum* accessions showed gene intersections with other groups, which might reflect from gene penetration in its breeding history.

The putative functional enrichment for all groups were assessed using EggNOG-mapper (v2.1.6)[39] based on EggNOG database (v5.0)[66]. The gene ontology enrichment analysis was carried out using AgriGO (v2.0)[67] and TBtools[68] (one-sided *p*-value by Fisher's exact test, adjusted by Benjamini-Hochberg correction), and the $-\log_{10}(p$-values) were illustrated by 'pheatmap' package in R (v3.6.0). In the functional enrichment, the unique core-genes and unique pan-genes for each genetic group were determined by removing genes shared between at least two groups. The GO enrichment of PAV clusters, pan-gene groups and unique pan-genes in genetic groups showed diverged functional enrichment between conserved genes (core and soft-core genes) and variable genes (shell and cloud genes). The conserved genes were enriched in functions such as "DNA-binding", "transcription regulation", "cell differentiation and communication", and "carbohydrate and lipid metabolic processes". The variable genes were enriched in functions such as "RNA-binding", "protein binding", "stress and stimulus response", "biosynthetic process" and "development". Notably, the unique genes of *P. abyssinicum* were found enriched in "stimulus and chemical response", while those of *P. fulvum* were enriched in "development", "growth", "reproduction", "cytoskeleton" and "tropism" (Supplementary Figure 16). These unique genes in *P. abyssinicum* and *P. fulvum* further confirmed their potential value as breeding materials to improve the resistance and production of pea cultivars in the future.

References

1. Koren, S. *et al.* Canu: scalable and accurate long-read assembly via adaptive k-mer weighting and repeat separation. *Genome Res.* **27**, 722-736 (2017).
2. Walker, B. J. *et al.* Pilon: an integrated tool for comprehensive microbial variant detection and genome assembly improvement. *PLoS One* **9**, e112963 (2014).
3. Roach, M. J., Schmidt, S. A. & Borneman, A. R. Purge Haplotigs: allelic contig reassignment for third-gen diploid genome assemblies. *BMC Bioinformatics* **19**, 460 (2018).
4. Yeo, S., Coombe, L., Warren, R. L., Chu, J. & Birol, I. ARCS: scaffolding genome drafts with linked reads. *Bioinformatics* **34**, 725-731 (2018).
5. Warren, R. L. *et al.* LINKS: Scalable, alignment-free scaffolding of draft genomes with long reads. *Gigascience* **4**, 35 (2015).
6. Durand, N. C. *et al.* Juicer provides a one-click system for analyzing loop-resolution Hi-C experiments. *Cell Syst.* **3**, 95-98 (2016).
7. Dudchenko, O. *et al.* De novo assembly of the Aedes aegypti genome using Hi-C yields chromosome-length scaffolds. *Science* **356**, 92-95 (2017).
8. Robinson, J. T. *et al.* Juicebox.js provides a cloud-based visualization system for Hi-C data. *Cell Syst.* **6**, 256-258 (2018).
9. Tang, H. *et al.* ALLMAPS: robust scaffold ordering based on multiple maps. *Genome Biol.* **16**, 3 (2015).
10. Tayeh, N. *et al.* Development of two major resources for pea genomics: the GenoPea 13.2K SNP Array and a high-density, high-resolution consensus genetic map. *Plant J.* **84**, 1257-1273 (2015).
11. Altschul, S. F., Gish, W., Miller, W., Myers, E. W. & Lipman, D. J. Basic local alignment search tool. *J. Mol. Biol.* **215**, 403-410 (1990).
12. Kent, W.J. BLAT--the BLAST-like alignment tool. *Genome Res.* **12**, 656-664 (2002).
13. Shen, W., Le, S., Li, Y. & Hu, F. SeqKit: A cross-platform and ultrafast toolkit for FASTA/Q file manipulation. *PLoS One* **11**, e0163962 (2016).
14. Smýkal, P. *et al.* From Mendel's discovery on pea to today's plant genetics and breeding. *Theor. Appl. Genet.* **129**, 2267-2280 (2016).
15. Kreplak, J. *et al.* A reference genome for pea provides insight into legume genome evolution. *Nat. Genet.* **51**, 1411-1422 (2019).
16. Pandey, A. K. *et al.* Omics resources and omics-enabled approaches for achieving high productivity and improved quality in pea (*Pisum sativum* L.). *Theor. Appl. Genet.* (2021).
17. Waterhouse, R. M. *et al.* BUSCO applications from quality assessments to gene prediction and phylogenomics. *Mo.l Biol. Evo.l* **35**, 543-548 (2018).
18. Rhie, A., Walenz, B. P., Koren, S. & Phillippy, A. M. Merqury: reference-free quality, completeness, and phasing assessment for genome assemblies. *Genome Biol.* **21**, 245 (2020).
19. Li, H. & Durbin, R. Fast and accurate short read alignment with Burrows-Wheeler transform. *Bioinformatics* **25**, 1754-1760 (2009).
20. Li, H. Minimap2: pairwise alignment for nucleotide sequences. *Bioinformatics* **34**, 3094-3100 (2018).
21. Ou, S., Chen, J. & Jiang, N. Assessing genome assembly quality using the LTR Assembly Index (LAI). *Nucleic. Acids Res.* **46**, e126 (2018).
22. Ellinghaus, D., Kurtz, S. & Willhoeft, U. LTRharvest, an efficient and flexible software for

de novo detection of LTR retrotransposons. *BMC Bioinformatics* **9**, 18 (2008).
23. Xu, Z. & Wang, H. LTR_FINDER: an efficient tool for the prediction of full-length LTR retrotransposons. *Nucleic Acids Res.* **35**, W265-W268 (2007).
24. Ou, S. & Jiang, N. LTR_retriever: a highly accurate and sensitive program for identification of long terminal repeat retrotransposons. *Plant Physiol.* **176**, 1410-1422 (2018).
25. Ou, S. & Jiang, N. LTR_FINDER_parallel: parallelization of LTR_FINDER enabling rapid identification of long terminal repeat retrotransposons. *Mobile DNA* **10**, 48 (2019).
26. Kim, D., Langmead, B. & Salzberg, S. L. HISAT: a fast spliced aligner with low memory requirements. *Nat. Methods* **12**, 357-360 (2015).
27. Pertea, M., Kim, D., Pertea, G. M., Leek, J. T. & Salzberg, S. L. Transcript-level expression analysis of RNA-seq experiments with HISAT, StringTie and Ballgown. *Nat. Protoc.* **11**, 1650-1667 (2016).
28. Niknafs, Y. S., Pandian, B., Iyer, H. K., Chinnaiyan, A. M. & Lyer, M. K. TACO produces robust multisample transcriptome assemblies from RNA-seq. *Nat. Methods* **14**, 68-70 (2017).
29. Haas, B. J. *et al.* Improving the *Arabidopsis* genome annotation using maximal transcript alignment assemblies. *Nucleic Acids Res.* **31**, 5654-5666 (2003).
30. Hoff, K. J., Lomsadze, A., Borodovsky, M. & Stanke, M. Whole-genome annotation with BRAKER. *Methods Mol. Biol.* **1962**, 65-95 (2019).
31. Gremme, G., Brendel, V., Sparks, M. E. & Kurtz, S. Engineering a software tool for gene structure prediction in higher organisms. *Inf. Softw. Technol.* **47**, 965-978 (2005).
32. Palmer, J. Funannotate: pipeline for genome annotation. https://funannotate.readthedocs.io/en/latest/index.html (2016).
33. Mistry, J., Finn, R. D., Eddy, S. R., Bateman, A. & Punta, M. Challenges in homology search: HMMER3 and convergent evolution of coiled-coil regions. *Nucleic Acids Res.* **41**, e121 (2013).
34. El-Gebali, S. *et al.* The Pfam protein families database in 2019. *Nucleic Acids Res.* **47**, D427-D432 (2019).
35. Consortium, T. U. UniProt: a worldwide hub of protein knowledge. *Nucleic Acids Res.* **47**, D506-D515 (2019).
36. Ogata, H. *et al.* KEGG: Kyoto encyclopedia of genes and genomes. *Nucleic Acids Res.* **27**, 29-34 (1999).
37. Ghosh, S. & Chan, C. K. Analysis of RNA-Seq data using TopHat and Cufflinks. *Methods Mol. Biol.* **1374**, 339-361 (2016).
38. Jones, P. *et al.* InterProScan 5: genome-scale protein function classification. *Bioinformatics* **30**, 1236-1240 (2014).
39. Cantalapiedra, C.P., Hernández-Plaza, A., Letunic, I., Bork, P. & Huerta-Cepas, J. eggNOG-mapper v2: Functional annotation, Orthology assignments, and Domain prediction at the metagenomic scale. *Mol. Biol. Evol.* (2021).
40. Liu, R. *et al.* Population genetic structure and classification of cultivated and wild pea (*Pisum* sp.) based on morphological traits and SSR markers. *J. Syst. Evol.* **60**, 85-100 (2022).
41. Danecek, P. *et al.* The variant call format and VCFtools. *Bioinformatics* **27**, 2156-2158 (2011).
42. Sedlazeck, F.J. *et al.* Accurate detection of complex structural variations using single-

molecule sequencing. *Nat. Methods* **15**, 461-468 (2018).

43. Smýkal, P. *et al.* Phylogeny, phylogeography and genetic diversity of the *Pisum* genus. *Plant Genet. Resour.* **9**, 4-18 (2010).

44. Zong, X. X. *et al.* Analysis of a diverse global *Pisum* sp collection and comparison to a Chinese local *P. sativum* collection with microsatellite markers. *Theor. Appl. Genet.* **118**, 193-204 (2009).

45. Sun, L. *et al.* *GmHs1-1*, encoding a calcineurin-like protein, controls hard-seededness in soybean. *Nat. Genet.* **47**, 939-943 (2015).

46. Wang, M. *et al.* Parallel selection on a dormancy gene during domestication of crops from multiple families. *Nat. Genet.* **50**, 1435-1441 (2018).

47. Chai, M. *et al.* A seed coat-specific β-ketoacyl-CoA synthase, KCS12, is critical for preserving seed physical dormancy. *Plant Physiol.* **186**, 1606-1615 (2021).

48. Bradbury, P. J. *et al.* TASSEL: software for association mapping of complex traits in diverse samples. *Bioinformatics* **23**, 2633-2635 (2007).

49. Broman, K. W., Wu, H., Sen, S. & Churchill, G. A. R/qtl: QTL mapping in experimental crosses. *Bioinformatics* **19**, 889-890 (2003).

50. Gonda, I. *et al.* Sequencing-based bin map construction of a tomato mapping population, facilitating high-resolution quantitative trait loci detection. *Plant Genome* **12**(2019).

51. Ellis, T. H., Hofer, J. M., Timmerman-Vaughan, G. M., Coyne, C. J. & Hellens, R. P. Mendel, 150 years on. *Trends. Plant Sci.* **16**, 590-596 (2011).

52. Reid, J. B. & Ross, J. J. Mendel's genes: toward a full molecular characterization. *Genetics* **189**, 3-10 (2011).

53. Barth, C. & Conklin, P.L. The lower cell density of leaf parenchyma in the *Arabidopsis thaliana* mutant *lcd1-1* is associated with increased sensitivity to ozone and virulent *Pseudomonas syringae*. *Plant J.* **35**, 206-218 (2003).

54. Shirasawa, K., Sasaki, K., Hirakawa, H. & Isobe, S. Genomic region associated with pod color variation in pea (*Pisum sativum*). *G3 (Bethesda, Md.)* **11**, jkab081 (2021)

55. Opassiri, R. *et al.* Analysis of rice glycosyl hydrolase family 1 and expression of Os4bglu12 beta-glucosidase. *BMC Plant Biol.* **6**, 33 (2006).

56. Clark, S.E., Running, M.P. & Meyerowitz, E.M. *CLAVATA1*, a regulator of meristem and flower development in *Arabidopsis*. *Development* **119**, 397-418 (1993).

57. Clark, S.E., Williams, R.W. & Meyerowitz, E.M. The *CLAVATA1* gene encodes a putative receptor kinase that controls shoot and floral meristem size in *Arabidopsis*. *Cell* **89**, 575-585 (1997).

58. Li, D., Liu, C. M., Luo, R., Sadakane, K. & Lam, T. W. MEGAHIT: an ultra-fast single-node solution for large and complex metagenomics assembly via succinct de Bruijn graph. *Bioinformatics* **31**, 1674-1676 (2015).

59. Zimin, A. V. *et al.* The MaSuRCA genome assembler. *Bioinformatics* **29**, 2669-2677 (2013).

60. Fu, L., Niu, B., Zhu, Z., Wu, S. & Li, W. CD-HIT: accelerated for clustering the next-generation sequencing data. *Bioinformatics* **28**, 3150-3152 (2012).

61. Alonge, M. *et al.* RaGOO: fast and accurate reference-guided scaffolding of draft genomes. *Genome Biol.* **20**, 224 (2019).

62. Glick, L. & Mayrose, I. Panoramic: A package for constructing eukaryotic pan-genomes. *Mol. Ecol. Resour.* **21**, 1393-1403 (2021).

63. Emms, D. M. & Kelly, S. OrthoFinder: phylogenetic orthology inference for comparative genomics. *Genome Biol.* **20**, 238 (2019).

64 Li H. *et al.* The Sequence Alignment/Map format and SAMtools. *Bioinformatics*. **25**: 2078-2079 (2009).

65. Page, A.J. *et al.* Roary: rapid large-scale prokaryote pan genome analysis. *Bioinformatics* **31**, 3691-3693 (2015).

66. Huerta-Cepas, J. *et al.* eggNOG 5.0: a hierarchical, functionally and phylogenetically annotated orthology resource based on 5090 organisms and 2502 viruses. *Nucleic Acids Res.* **47**, D309 - D314 (2019).

67. Tian, T. *et al.* agriGO v2.0: a GO analysis toolkit for the agricultural community, 2017 update. *Nucleic Acids Res.* **45**, W122-W129 (2017).

68. Chen, C. *et al.* TBtools: An integrative toolkit developed for interactive analyses of big biological data. *Mol. Plant.* **13**, 1194-1202 (2020).
