# Supplementary material for: Improved pea reference genome and pan-genome highlight genomic features and evolutionary characteristics
Source: Nat Genet. 2022 Sep 22;54(10):1553–63. doi: 10.1038/s41588-022-01172-2 (PMC9534762; doi:10.1038/s41588-022-01172-2)
Supplement: Supplementary file 2 — Reporting Summary [file 41588_2022_1172_MOESM2_ESM.pdf]

nature portfolio

Corresponding author(s): Xuxiao Zong

Last updated by author(s): Jul 20, 2022

Reporting Summary

Nature Portfolio wishes to improve the reproducibility of the work that we publish. This form provides structure for consistency and transparency in reporting. For further information on Nature Portfolio policies, see our Editorial Policies and the Editorial Policy Checklist.

Statistics

For all statistical analyses, confirm that the following items are present in the figure legend, table legend, main text, or Methods section.

n/a	Confirmed	
☐	☒	The exact sample size (*n*) for each experimental group/condition, given as a discrete number and unit of measurement
☐	☒	A statement on whether measurements were taken from distinct samples or whether the same sample was measured repeatedly
☐	☒	The statistical test(s) used AND whether they are one- or two-sided<br>*Only common tests should be described solely by name; describe more complex techniques in the Methods section.*
☒	☐	A description of all covariates tested
☐	☒	A description of any assumptions or corrections, such as tests of normality and adjustment for multiple comparisons
☐	☒	A full description of the statistical parameters including central tendency (e.g. means) or other basic estimates (e.g. regression coefficient) AND variation (e.g. standard deviation) or associated estimates of uncertainty (e.g. confidence intervals)
☐	☒	For null hypothesis testing, the test statistic (e.g. *F*, *t*, *r*) with confidence intervals, effect sizes, degrees of freedom and *P* value noted<br>*Give P values as exact values whenever suitable.*
☒	☐	For Bayesian analysis, information on the choice of priors and Markov chain Monte Carlo settings
☒	☐	For hierarchical and complex designs, identification of the appropriate level for tests and full reporting of outcomes
☒	☐	Estimates of effect sizes (e.g. Cohen's *d*, Pearson's *r*), indicating how they were calculated

Our web collection on statistics for biologists contains articles on many of the points above.

Software and code

Policy information about availability of computer code

Data collection	No commercial and custom code was used for data collection.
Data analysis	Genome size estimation was performed using FloMax (v2.3) and JellyFish (v2.3.0). Genome assembly and assessment were performed using: Canu (v1.8), Pilon (v1.23), PurgeHaplotigs (v1.1.1), ARCS (v1.0.4), LINKS (v1.8.6), Bionano Solve package (v3.4_06042019a), Juicer (v1.5.6), 3d-dna pipeline (v180922), Juicebox (v1.11.08), ALLMAPS (v1.0), BLAST (v2.5.0+), BLAT (v34), SeqKit (v0.15.0), Merqury (v1.3), BWA-MEM (v0.7.15) and minimap2 (v2.1), LTRharvest in GenomeTools(v1.6.0), LTR_FINDER (v1.0.7), LTR_retriever(v2.9.0). Genome annotation and expression analysis were performed using: RepeatMasker (v4.1.1), LTR_FINDER_parallel (v1.0.7), HISAT2 (v2.1.0), StringTie (v1.3.4), TACO (v0.7.3), TransDecoder (v5.5.0), BRAKER2 pipeline (v2.1.5), GenomeThreader (v1.7.1), Funannotate (v1.7.4), HMMER (v3.3.1), BLASTP (v2.5.0+), GFFRead in Cufflinks (v0.11.6), InterProScan (v5.0), eggNOG-mapper (v2.1.6), Trimmomatic(v0.39), FastQC (v 0.11.9). Comparative genome analysis was performed using: MCScanX, and OrthoFinder (v2.3.12). SNV and SV analyses were performed using: GATK (v4.1), VCFtools (v0.1.15), snpEff (v4.3t), Delly (v0.8.3), BCFtools (v1.8), FastTree (v2.1.10), FigTree (v1.4.3), PLINK (v1.90b4.6), ADMIXTURE (v1.3.0), PopLDdecay, and XP-CLR. QTL analyses were performed using: SPSS (v16.0), Tassel (v5.2.40), R/qtl, SNPbinner, R (v3.6.0) packages (LinkageMapView and CMplot). Pan-genome assembly, gene annotation and clustering were performed using: MEGAHIT (v1.2.9), MaSuRCA (v3.4.0), MUMmer (v4.0), CD-HIT (v4.8.1), RagTag (v2.0.1), minimap2 (v2.1), samtools (v1.12), minigraph (v0.13), gfatools(v0.5), BRAKER2, HMMER (3.3.1), BLASTP (2.5.0+), OrthoFinder (v2.3.12), R (v3.6.0) packages (pheatmap, hclust, reshape2, RColorBrewer, randomForest, Boruta, pROC, caret, and ggplot2), EggNOG-mapper (v2.1), AgriGO (v2.0), and TBtools (v1.0692). Construction of phylogenetic tree were performed using: BLAST (v2.9.0), MEGA (v7.0). Other basic statistics and illustrations were performed using SeqKit (v0.15.0) and TBtools (v1.0692). The custom scripts used in PeaZW6 genome and pan-genome project have been deposited in Zenodo (https://doi.org/10.5281/zenodo.6614849).

For manuscripts utilizing custom algorithms or software that are central to the research but not yet described in published literature, software must be made available to editors and reviewers. We strongly encourage code deposition in a community repository (e.g. GitHub). See the Nature Portfolio guidelines for submitting code & software for further information.

Data

Policy information about availability of data

All manuscripts must include a data availability statement. This statement should provide the following information, where applicable:

- Accession codes, unique identifiers, or web links for publicly available datasets
- A description of any restrictions on data availability
- For clinical datasets or third party data, please ensure that the statement adheres to our policy

This Whole Genome Shotgun project of Pisum sativum cultivar Zhongwan6 (PeaZW6) has been deposited at DDBJ/ENA/GenBank under the accession JAMSHJ000000000. All raw sequencing data and the 118 pan-genome assemblies has been deposited at NCBI under the BioProject PRJNA730094. The PeaZW6 assembly (https://doi.org/10.5281/zenodo.6622409) and the 118 pan-genome assemblies (https://doi.org/10.5281/zenodo.6622578) are also available under Zenodo datasets. The PeaZW6 assembly along with genome browser and basic analysis tools are also available at Pea Genome Database (https://www.peagdb.com/).

Field-specific reporting

Please select the one below that is the best fit for your research. If you are not sure, read the appropriate sections before making your selection.

☒ Life sciences ☐ Behavioural & social sciences ☐ Ecological, evolutionary & environmental sciences

For a reference copy of the document with all sections, see nature.com/documents/nr-reporting-summary-flat.pdf

Life sciences study design

All studies must disclose on these points even when the disclosure is negative.

Sample size	Samples were chose to fully representing cultivated and wild pea accessions to obtain meaningful results.
Data exclusions	No data were excluded from analysis in this study.
Replication	Three distinct sample repetitions for the standard sample tomato and the Chinese pea cultivar ZW6 were used in flow cytometry experiment to estimate the genome size of PeaZW6 (Supplementary Figure 2).
Randomization	Plants were randomly allocated in the glasshouse.
Blinding	This is not relevant for the method, because it does not depend on the statistical variation of the properties of the samples.

Reporting for specific materials, systems and methods

We require information from authors about some types of materials, experimental systems and methods used in many studies. Here, indicate whether each material, system or method listed is relevant to your study. If you are not sure if a list item applies to your research, read the appropriate section before selecting a response.

Materials & experimental systems

n/a	Involved in the study	
☒	☐	Antibodies
☒	☐	Eukaryotic cell lines
☒	☐	Palaeontology and archaeology
☒	☐	Animals and other organisms
☒	☐	Human research participants
☒	☐	Clinical data
☒	☐	Dual use research of concern

Methods

n/a	Involved in the study	
☒	☐	ChIP-seq
☐	☒	Flow cytometry
☒	☐	MRI-based neuroimaging

Flow Cytometry

Plots

Confirm that:

☒ The axis labels state the marker and fluorochrome used (e.g. CD4-FITC).

☒ The axis scales are clearly visible. Include numbers along axes only for bottom left plot of group (a 'group' is an analysis of identical markers).

☒ All plots are contour plots with outliers or pseudocolor plots.

☒ A numerical value for number of cells or percentage (with statistics) is provided.

Methodology

Sample preparation	Sample were placed in 500 ul Nuclei Extraction buffer, chopped with sharp blade then filtered through a 50 um filter after 60 seconds. Followed by addition of 2000 ul of staining buffer with RNase for 30 minutes in dark.
Instrument	CyFlow Space Flow Cytometer (Sysmex Partec GmbH, Muenster, Germany).
Software	FloMax software (Sysmex Partec GmbH, Muenster, Germany).
Cell population abundance	Intact cells account for more than 85% of the total number of collected data.
Gating strategy	N/A

☐ Tick this box to confirm that a figure exemplifying the gating strategy is provided in the Supplementary Information.
